# Induction of autophagy in Cx3cr1^+^ mononuclear cells limits IL-23/IL-22 axis-mediated intestinal fibrosis

**DOI:** 10.1038/s41385-019-0146-4

**Published:** 2019-02-14

**Authors:** Ramkumar Mathur, Mahabub Maraj Alam, Xiao-Feng Zhao, Yuan Liao, Jeffrey Shen, Shannon Morgan, Tingting Huang, HwaJeong Lee, Edward Lee, Yunfei Huang, Xinjun Zhu

**Affiliations:** 1 Department of Molecular and Cellular Physiology, Albany Medical College, Albany, NY 12208, USA; 2 The IBD Center, Division of Gastroenterology, Department of Medicine, Albany Medical College, Albany, NY 12208, USA; 3 Department of Neuroscience and Experimental Therapeutics, Albany Medical College, Albany, NY 12208, USA; 4 Department of Pathology, Albany Medical College, Albany, NY 12208, USA; 5 Department of Surgery, Albany Medical College, Albany, NY 12208, USA

## Abstract

Intestinal fibrosis is an excessive proliferation of myofibroblasts and deposition of collagen, a condition frequently seen in Crohn’s disease (CD). The mechanism underlying myofibroblast hyper-proliferation in CD needs to be better understood. In this report, we found that mTOR inhibitor rapamycin or mTOR deletion in CX3Cr1^+^ mononuclear phagocytes inhibits expression of interleukin (IL) −23, accompanied by reduced intestinal production of IL-22 and ameliorated fibrosis in the TNBS-induced fibrosis mouse model. This inhibition of IL-23 expression is associated with elevated autophagy activity. Ablating the autophagy gene Atg7 increases the expression of IL-23, leading to increased expression of IL-22 and increased fibrosis. Both induction of IL-22 and intestinal fibrosis occurred in RAG^−/−^ mice and depletion of innate lymphoid cells (ILCs) attenuates the fibrotic reaction, suggesting that the pro-fibrotic process is independent of T and B cells. Moreover, IL-22 facilitates the transformation of fibroblasts into myofibroblasts. Finally, the fibrotic reaction was attenuated upon neutralization of either IL-23 or IL-22. Altogether, this study elucidated a signaling cascade underlying intestinal fibrosis in which altered mTOR/autophagy in CX3Cr1^+^ mononuclear phagocytes up-regulates the IL-23/IL-22 axis, leading to an excessive fibrotic response. Thus, our findings suggest that this cascade could be a therapeutic target for alleviation of CD fibrosis.

## INTRODUCTION

Intestinal fibrosis is a severe complication of inflammatory bowel diseases (IBDs) such as Crohn’s disease (CD), and is commonly revealed as intestinal stricture or stenosis.^1^ Fibrosis gradually evolves in response to prolonged intestinal injury or inflammation, but its manifestation does not necessarily correlate with the severity of inflammation. Fibrosis is generally considered to be irreversible. Despite the advent of new therapeutics (biologics) for IBDs, the incidence of stricture formation and stenosis of the intestine in IBD patients has not improved significantly.

Autophagy is a highly conserved catabolic pathway which assists in the sequestration and removal of unwanted cellular debris.^2^ Impaired autophagy is associated with the risk of development of CD.^3,4^ Genome-wide association studies (GWAS) have shown that more than 200 genes or loci are associated with a high risk of IBD. Mutation of genes in the autophagy pathway, including ATG16L1,^3,5^ NOD2,^6,7^ IRGM,^8,9^ LRRK2,^10,11^ and ULK1,^12^ predisposes to severe fibrotic CD. Recent studies suggest that autophagy regulates intracellular degradation of type I collagen.^13^ Treatment with rapamycin, a pharmacological inhibitor of mTOR, activates autophagy and reduces active colitis, IPEX (Immune dysregulation, polyendocrinopathy, enteropathy, X-linked syndrome) and IPEX-like enteropathy in children.^14,15^ Conversely, inactivation of the autophagy pathway causes accumulation of type I collagen and promotes fibrosis in kidney.^16^ However, whether autophagy inactivation has the same promoting-effect in intestinal fibrosis remains unclear.

Interleukin (IL)-23 secreted from macrophages and dendritic cells acts as a pleiotropic cytokine. IL-23 has been shown to induce secretion of both IL-17 and IL-22 from T cells^17–20^ and innate lymphoid cells (ILCs; e.g., ILC3).^21,22^ IL-23/IL-23-receptor-mediated induction of the IL-17 and IL-22 pathways has gained significant attention in recent years because of their leading roles in gut immunity and tissue repair.^23–25^ Furthermore, GWAS findings revealed that the IL-23R gene is a risk factor in IBD.^11,26,27^ Genetic deletion or neutralization of IL-23 reduces IL-17 accumulation and ameliorates intestinal inflammation.^28^ Mice deficient for IL-23p19 are more susceptible to colitis in the experimental T cell-mediated TNBS model.^28^ The level of IL-17 is elevated in the intestine of IBD patients, where the cytokine facilitates intestinal fibrosis.^24,29^ Likewise, IL-23 induced-expression of IL-22 is observed in psoriasis,^30^ rheumatoid arthritis,^31^ and IBD.^32,33^ CX3Cr1^+^ mononuclear phagocytes promote the production of IL-22 from ILC3 cells via IL-23.^22,34^ IL-23-deficient mice are susceptible to *Citrobacter rodentium* infection but can be rescued by treatment with exogenous recombinant IL-22, which presumably boosts the production of antimicrobial peptides or promotes proliferation and survival of epithelial progenitors and tissue repair.^34,35^ Mice with depletion of ILC3 cells display impaired induction of IL-22 and become more susceptible to bacterial-induced severe colitis.^22^ However, IL-23/IL-22 was also reported to exacerbate the inflammation in a chronic/adaptive colitis model, reflecting the complexity of this axis in IBD pathogenesis.^32,36^

Intestinal fibrosis, to some extent, is an exaggerated repair process in response to inflammation and injury. Although it is well documented that TGFβ, a key cytokine produced by Cx3cr1^+^ mononuclear phagocytes, is involved in intestinal fibrosis, the role of the macrophage-mediated IL-23/IL-22 axis in that pathology remains unclear. IL-22 promotes intestinal epithelial regeneration and wound healing.^32,36,37^ Thus, it is conceivable that the IL-23/IL-22 axis plays a role in intestinal fibrosis. A recent study reported that IL-22 regulates the fibrotic reaction in acute skin wounding.^23^ However, IL-22 secreted from γδT cells was reported to inhibit lung fibrosis,^38^ suggesting that IL-22 regulates fibrosis in a tissue-specific manner. Therefore, the role of IL-22 in intestinal fibrosis needs to be evaluated further.

In this study, we sought to determine the role of the IL-23/IL-22 axis promoted by CX3CR1^+^ mononuclear phagocytic cells in intestinal fibrosis. We also clarified the role of the autophagy pathway in regulation of the IL-23/IL-22 axis.

## RESULTS

### Induction of IL-23/IL-22 along with intestinal fibrosis in the TNBS model of inflammatory bowel disease

**Fig. 1:**
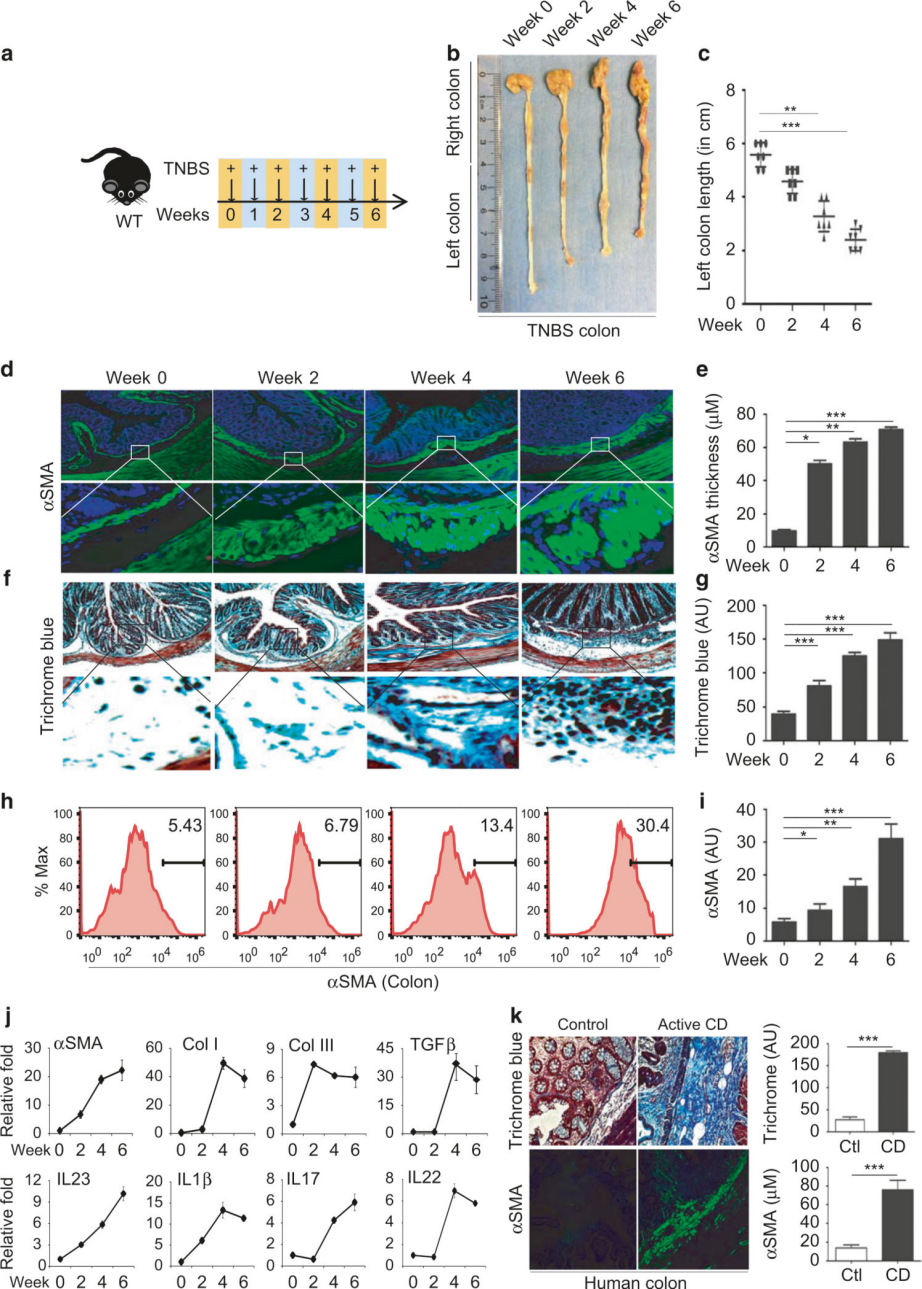
Serial TNBS rectal administration promotes the development of intestinal fibrosis. **a** Schematic diagram showing the regimen of weekly TNBS treatment given to wild-type mice; **b** Representative images of colon harvested on week 0, 2, 4, and 6 post-TNSB treatment; **c** Average length of distal colon, called left colon, plotted in the graph (*n*= 4–6 mice per group); histological analysis of colon fibrosis shown by representative images of myofibroblasts stained with anti-αSMA antibody (**d**) and collagen stained by Trichrome blue (**f**); **e** Quantification of the thickness of the submucosal αSMA-positive layer and average intensity of Trichrome blue staining at week 0, 2, 4, and 6 (**g**); **h** FACS analysis of αSMA-positive cells in the colon, and quantification of αSMA-positive cells (**i**); qPCR analysis of fibrosis markers and cytokines (**j**); Data are presented as mean ± SEM (*t*-test). *n*= 3–6/group. **k** Histological analysis of myofibroblast proliferation with αSMA staining and trichrome blue staining for collagen deposition in colon tissue resections from Crohn’s disease patients and controls. Quantification of the intensity of trichrome blue and submucosal αSMA staining in three pairs of surgical specimens. *N*= 3 *p < 0.05, ^**^p < 0.01, ^***^p < 0.001

Proliferation of αSMA-positive myofibroblasts and collagen deposition are well-defined histological changes in Crohn’s fibrosis.^4,39^ To better elucidate the fibrogenic mechanism, we adopted the TNBS mouse model of fibrosis, which has been frequently used to model intestinal fibrosis of CD.^40^ We began with a detailed characterization of TNBS time kinetics in wild-type mice by weekly administration of TNBS rectally for up to 6 weeks, as indicated schematically in Fig. 1a. We observed progressive shortening of the left colon over the course of TNBS treatment, from an average of 5 ± 0.5 cm in the control group to 3 ± 0.5 cm in the TNBS group 6 weeks post-treatment (Fig. 1b, c). Mice also displayed significant weight loss, mainly around weeks 4 and 6 (Fig S1a). Histological analysis revealed a 4–6-fold increase in the thickness of the colonic submucosa layer positively stained with αSMA (Fig. 1d, e). Trichrome blue staining revealed increased deposition of collagen by 2–4-fold within the submucosal layers (Fig. 1f, g), confirming severe intestinal fibrosis. FACS analysis demonstrated a significant accumulation of αSMA-positive cells in the colon of TNBS-treated mice, suggesting hyperactivation of myofibroblasts (Fig. 1h, i). Western blot analysis revealed increased expression of αSMA protein (Fig S1b & S1c). Moreover, there was marked induction of αSMA, Col-I, and Col-III, along with TGFβ1, a classic pro-fibrotic cytokine (Fig. 1j). Apart from the fibrotic changes, we also detected moderate induction of IL-1β, IL-23, IL-17, and IL-22 over the course of TNBS treatment (Fig. 1j), suggesting moderate induction of inflammation. We also investigated fibrotic reactions in colon resections from patients newly diagnosed with CD. We observed significant thickening of submucosal layers filled with αSMA-positive myofibroblasts and significant deposition of collagen in CD compared to controls (Fig. 1k) (Supplementary Table 1). These results suggest that the fibrotic reaction occurs relatively earlier than previously thought and likely parallels intestinal inflammation.

We performed additional analysis of fibrosis markers and cytokines in fresh biopsy tissue from the ilea of patients with active CD or under remission. We observed increased collagen deposition and thickening of aSMA-positive layers in active CD (Fig S2a–S2b) (Supplementary Table 2). Western blot analysis confirmed increased αSMA in active CD (Figs. S2c–2d). There was significant induction of fibrosis markers including αSMA, Col1, and TGFβ1, and cytokines, including IL-1β, IL-23, IL-22, IL-17, TNFα, IFNγ, and IFNβ (Fig S2e).

### Rapamycin treatment suppresses IL-23/IL-22 expression and ameliorates fibrosis

**Fig. 2:**
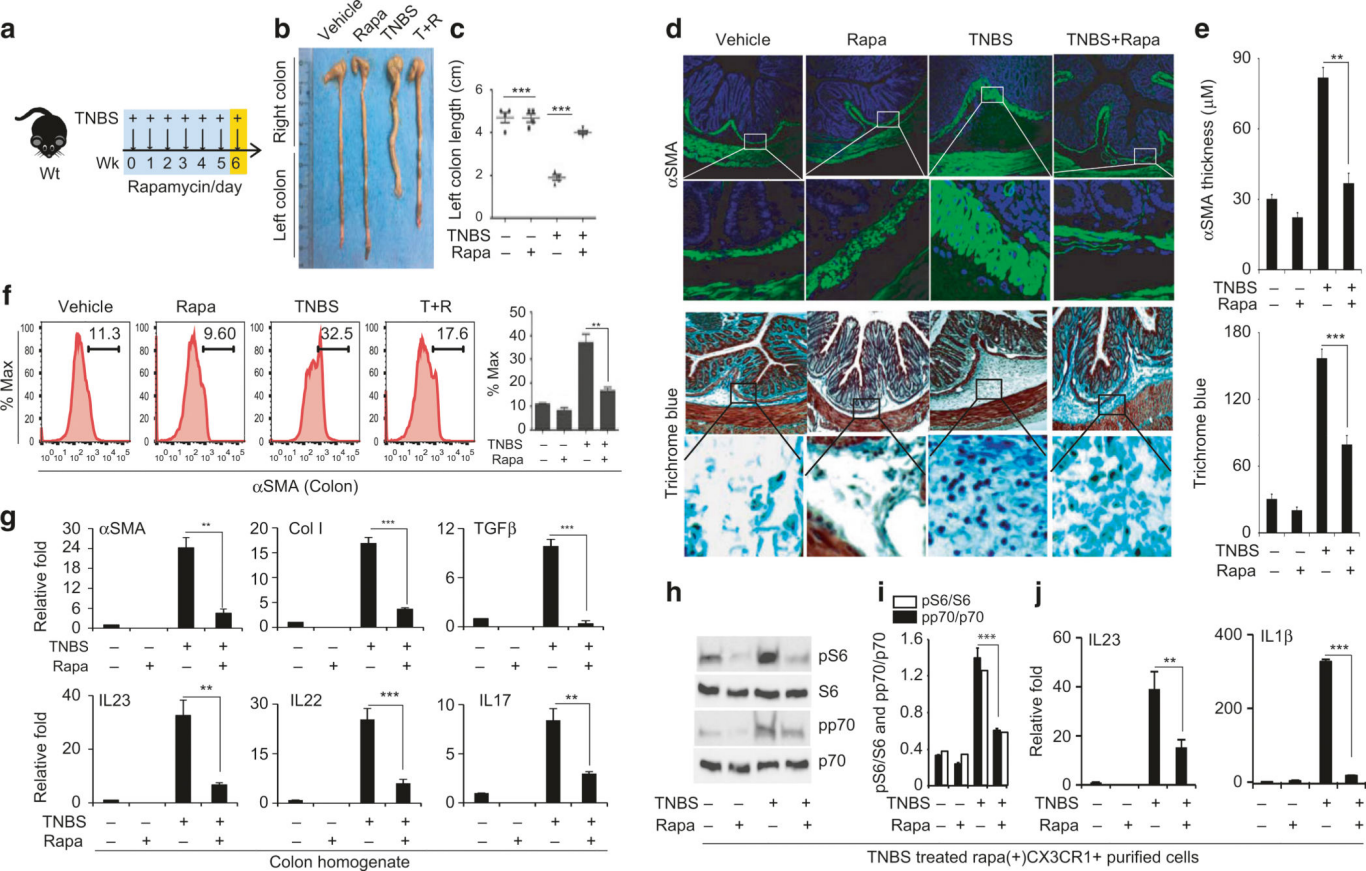
Rapamycin effectively ameliorates fibrosis and suppresses induction of IL-23/IL-22 expression. **a** Schematic diagram showing the treatment regimen of TNBS administered weekly and rapamycin given daily on weekdays to wild-type mice; **b**, **c** Representative images of colon harvested on week 6 post-TNBS and/or after rapamycin treatment, and the average length of the left colon (*n*= 5–7 mice each group); **d** Histological analysis of colon fibrosis show representative images of myofibroblast staining with anti-αSMA antibody and collagen staining with Trichrome blue; **e** Quantification of the thickness of the submucosal αSMA-positive layer and average intensity of Trichrome blue staining; **f** FACS analysis of αSMA-positive cells in the colon and quantification of αSMA-positive cells; **g** qPCR analysis of fibrosis markers and cytokines; **h** Western blot analysis of p-p70 and p-S6 levels in purified Cx3cr1^+^ mononuclear phagocytes from mice treated with TNBS and/or rapamycin; **i** quantification of p-p70 (open bar) and p-S6 (closed bar) levels; **j** qPCR analysis of the expression of IL-23 and IL-1β in purified Cx3cr1^+^ mononuclear phagocytes; Data are presented as mean ± SEM. *n*= 5. *p < 0.05, ^**^p < 0.01, ^***^p < 0.001

We set out to evaluate the effect of TNBS treatment on mTOR activity. We observed a moderate increase in the levels of phosphorylated p70 and S6 in the colons of mice treated with TNBS (Figs. S1d–1f), indicative of mTOR activation. Accordingly, we treated mice with both TNBS and the mTOR inhibitor rapamycin as illustrated in the schematic diagram (Fig. 2a). Rapamycin attenuated the weight loss (data not shown) and partially abrogated the colon shortening (Fig. 2b, c). Furthermore, colon histology showed that rapamycin prevented thickening of the αSMA-positive submucosal layer and reduced collagen deposition there (Fig. 2d, e). FACS analysis revealed that the αSMA^−^positive population was significantly lower in the colons of mice treated with both rapamycin and TNBS, compared with the TNBS-treated group (Fig. 2f). Moreover, rapamycin effectively prevented the induction of the fibrosis markers αSMA, Col I and Col III as well as the cytokines IL-23, IL-1β, IL-17, and TGFβ in colon tissue homogenates (Fig. 2g & S3a).

Mononuclear phagocytes are the principal cells that mediate the innate immune response to injury. Having demonstrated activation of mTOR and induction of cytokines in colon homogenate, we next determined whether this reflects changes in mononuclear phagocytes. We performed FACS analysis of Cx3cr1^+^ resident mononuclear phagocytes in the colon tissue. We observed significant upregulation of MHC-II-, CD40-, CD80-, and CD86-positive cells in the colons prepared from mice treated with TNBS for 6 weeks, indicating activation of macrophages. Moreover, this change was blocked by rapamycin treatment (Fig S3b). We purified CX3CR1^+^ resident mononuclear phagocytes using magnetic microbeads (Fig S3c–S3d). Western blot analysis revealed increased levels of p-p70 and p-S6 in CX3CR1^+^ resident mononuclear phagocytes prepared from TNBS-treated mice, which was blocked by rapamycin (Fig. 2h, i). We further confirmed there was significant induction of IL-23 and IL-1β in the TNBS-treated group (Fig. 2j). This induction was also attenuated by rapamycin. Together, our data demonstrate that, in the TNBS model, activation of mTOR induced the expression of IL-23 and IL-1β in Cx3cr1^+^ mononuclear phagocytes and promoted intestinal fibrosis. *We further characterized CD64*^+^*/CD11c*^-^*/Cx3cr1*^+^ *cells according to previous studies*^34,41–43^ (Fig S4a–S4c). We observed a significant increase of the MHCII^high^/Cx3cr1^int^/Ly6C^high^(P2) population, with a moderate reduction of the MHCII^high^/Cx3cr1^int^/Ly6C^low^(P3) and MHCII^high^/Cx3cr1^hi^/Ly6c^low^ (P4) populations in the TNBS-treated group. qPCR analysis revealed elevated levels of inflammatory cytokines, including IL1β, TNFα, IL23, and IL22, and reduced levels of IL10 in the P3 and P4 populations (Fig S4d).

### Rapamycin inhibits the induction of IL-23 and IL-1β via activation of autophagy in bone marrow-derived macrophages

**Fig. 3:**
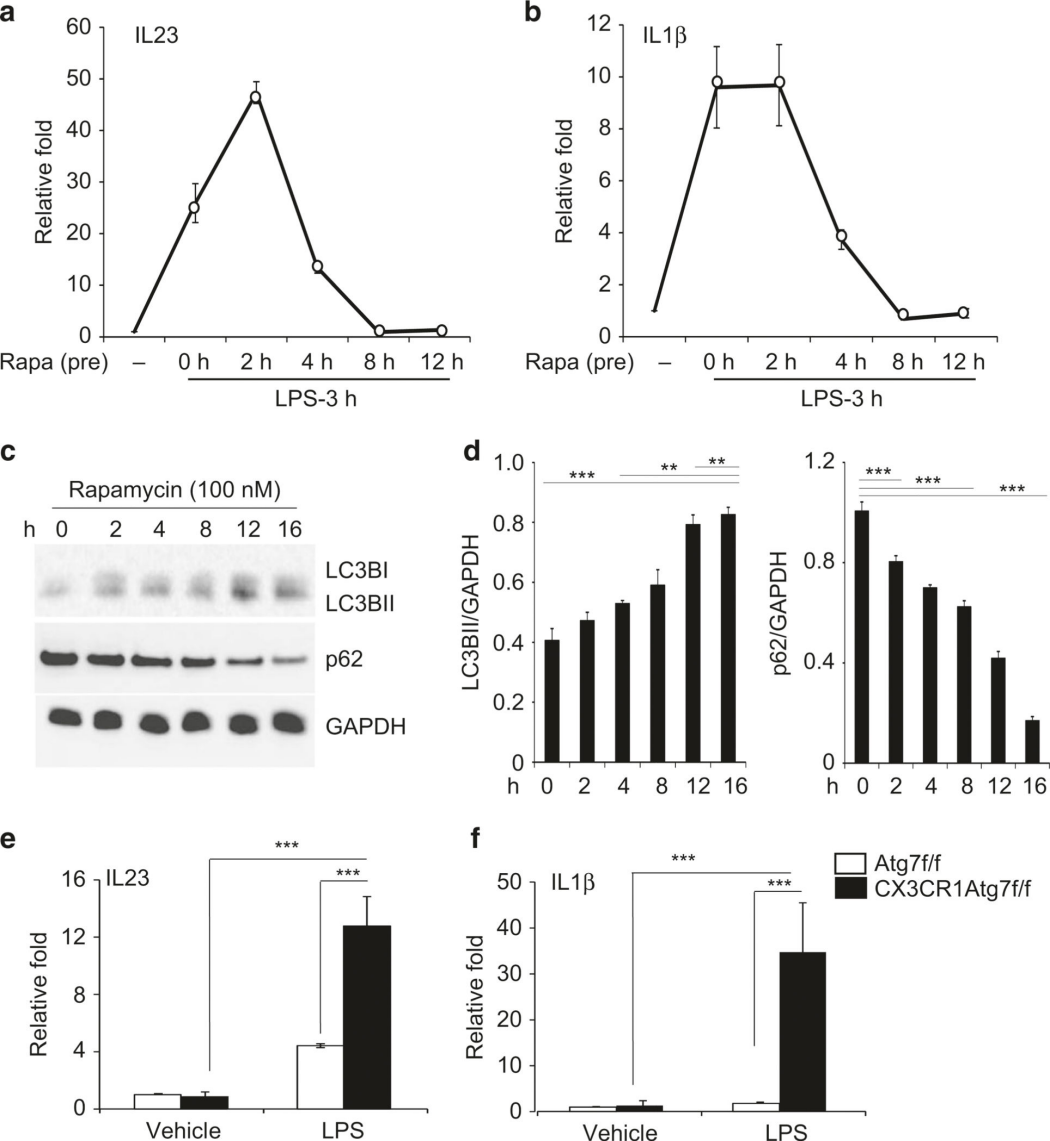
Induction of autophagy limits induction of IL-23 and Il-1β in macrophages. Bone marrow-derived macrophages (BMDMs) prepared from wild-type mice were pre-incubated with rapamycin at 100 nM for 2–12 h and then stimulated with LPS (10 ng/ml) for 3 h. qPCR analysis for the expression of IL-23 (**a**) and IL-1β (**b**); **c** Western blot analysis for the levels of LC3B-1/B-II, p62 and GAPDH in BMDMs treated with rapamycin at 100 nM for 2–18 h; **d** Quantification of LC3B-1/II and p62 in comparison to GAPDH control via densitometry; **e**, **f** BMDMs were prepared from control (Atg7^f/f^) and Cx3cr1-cre;Atg7^f/f^ mice and stimulated with LPS (10 ng/ml) for 3 h. qPCR analysis for the expression of IL-23 and IL-1β; Data are presented as mean ± SEM (*t*-test) of at least *n*= 4 experiments, ^∗^*p* < 0.05, ^∗∗^*p* < 0.01, ^∗∗∗^*p* < 0.001

We next evaluated the effect of rapamycin on IL-23 expression in bone marrow-derived macrophages (BMDMs). BMDMs were first exposed to rapamycin at 100 nM for 2–12 h prior to treatment with lipopolysaccharide (LPS). LPS stimulated a 25-fold induction of IL-23 transcription in BMD macrophages (Fig. 3a). The induction was nearly doubled in the group that was pre-treated with rapamycin for 2 h. However, prolonged exposure to rapamycin for 8–12 h suppressed the induction. Likewise, induction of IL-1β was also suppressed after prolonged exposure to rapamycin (Fig. 3b).

Inhibition of mTOR is known to stimulate autophagy activity.^44,45^ Indeed, we observed a significant increase in LC3B-II/LC3BI protein and reduced levels of p62, suggesting that autophagy activity increases in BMDMs following prolonged treatment with rapamycin (Fig. 3c, d). To ascertain that autophagy regulates cytokine induction, we performed LPS stimulation in BMDMs prepared from control and Cx3cr1-cre: Atg7^f/f^ mice. We found that deletion of Atg7 significantly boosted the induction of IL-23 and IL-1β transcription (Fig. 3e, f). Thus, activation of autophagy upon prolonged exposure to rapamycin limits the LPS-induced inflammatory response.

### Inactivation of autophagy in Cx3cr1^+^ mononuclear phagocytes aggravates the inflammatory response and exacerbates fibrosis in the TNBS model

**Fig. 4:**
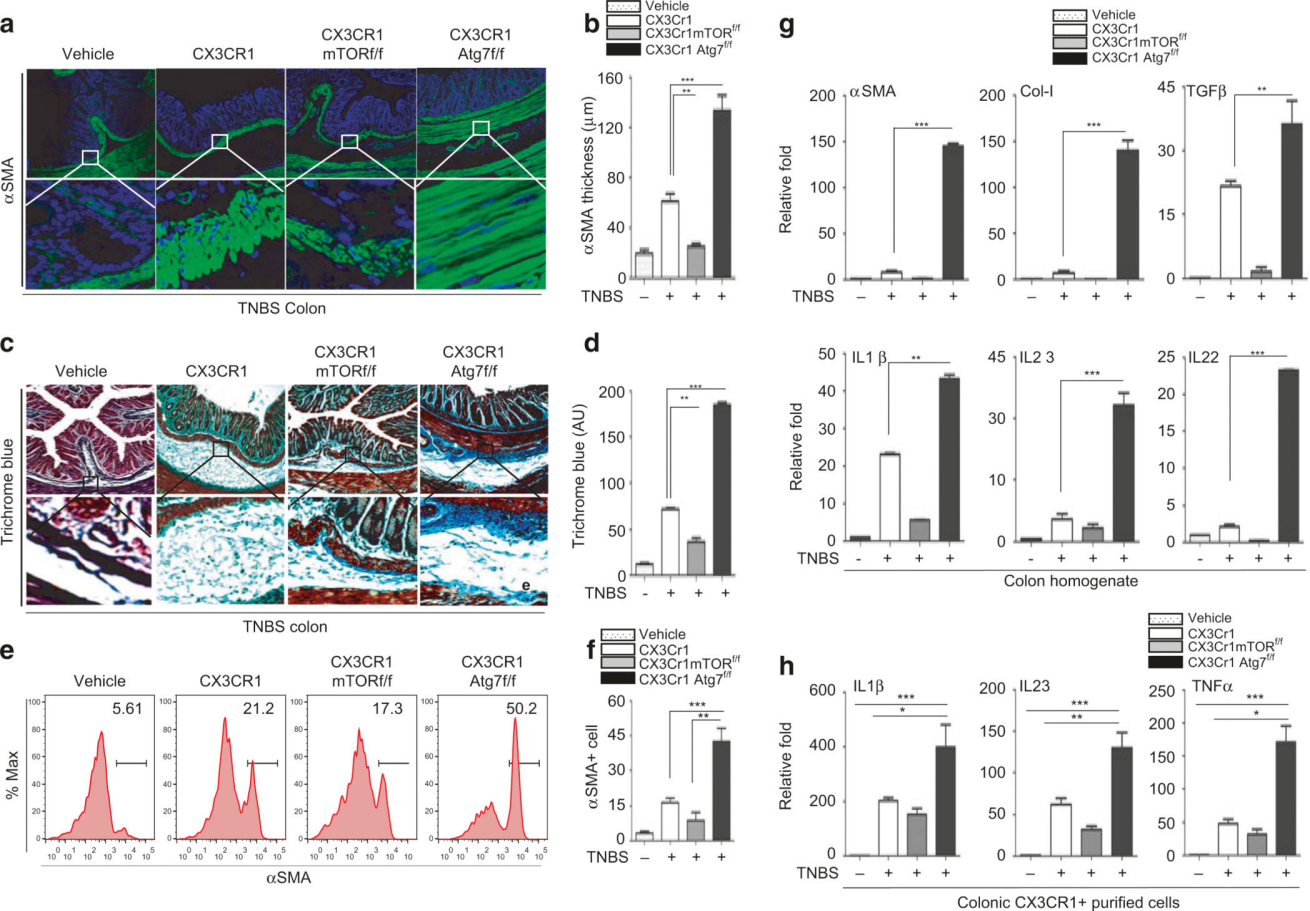
Inflammatory and fibrotic responses in Cx3cr1-cremTOR^f/f^ and Cx3cr1-creAtg7^f/f^ mice. Histological analysis of colon fibrosis in CX3cr1, CX3cr1mTOR^f/f^, and CX3cr1Atg7^f/f^ mice. Representative images of myofibroblast staining with anti-αSMA antibody (**a**) and collagen staining with Trichrome blue (**c**); Quantification of the thickness of the submucosal αSMA-positive layer (**b**) and average intensity of Trichrome blue staining (**d**); **e**, **f** FACS analysis and quantification of αSMA-positive cells in the colon; **g** qPCR analysis of fibrosis markers and cytokines; **h** Expression of IL-23, IL-1β, and TNFα in purified CX3Cr1^+^ mononuclear phagocytes in mouse colon samples measured by qPCR. Data are presented as mean ± SEM. *n*= 5, *p < 0.05, ^**^p < 0.01, ^***^p < 0.001

We next determined whether the mTOR/autophagy pathway in Cx3cr1^+^ mononuclear phagocytes regulates the inflammatory response and intestinal fibrosis in the TNBS model. We employed Cx3cr1-cre; mTOR^f/f^ and Cx3cr1-cre; Atg7^f/f^ mice in which mTOR and Atg7 were deleted in Cx3cr1^+^ cells. Animals were treated with TNBS for 6 weeks and intestinal tissues were harvested at week 6 (Fig S5a). Colon shortening was moderate in TNBS-treated Cx3cr1-cre:mTOR^f/f^ mice compared with TNBS-treated wild-type mice; however, it was exacerbated in Cx3cr1-cre:Atg7^f/f^ mice (Fig S5b & S5c). The thickness of the αSMA-positive submucosal layer in TNBS-treated Cx3cr1-cre:mTOR^f/f^ mice was much less than that in the colon of TNBS-treated wild-type mice (Fig. 4a, b). In contrast, the thickness of the αSMA-positive submucosal layer was significantly increased in Cx3cr1-cre:Atg7^f/f^ mice. Likewise, trichrome blue staining revealed reduced collagen deposition in the submucosal layer of Cx3cr1-cre:mTOR^f/f^ mice, whereas collagen deposition was increased in Cx3cr1-cre:Atg7^f/f^ mice compared to TNBS-treated wild-type mice (Fig. 4c, d). Additionally, FACS analysis revealed that the population of αSMA-positive cells was reduced in TNBS-treated Cx3cr1-cre:mTOR^f/f^ mice compared with wild-type mice. In contrast, there was significant expansion of αSMA-positive cells in the colon of Cx3cr1-cre:Atg7^f/f^ mice (Fig. 4e, f). Consistent with the histology, gene-expression of the fibrosis markers collagen and αSMA was reduced in Cx3cr1-cre:mTOR^f/f^ mice, whereas it was elevated in Cx3cr1-cre:Atg7^f/f^ mice compared with wild-type mice treated with TNBS (Fig. 4g). These data are consistent with the observed histological changes. The transcription levels of IL-23, IL-1β, IL-22, and TGFβ in mouse colon were significantly higher in Cx3cr1-cre:Atg7^f/f^ mice than in wild-type and Cx3cr1-cre:mTOR^f/f^ mice (Fig. 4g, S5d & S5g). Moreover, FACS analysis revealed that the levels of IL-23 and IL-1β in the CD11b + /F4/80 + population in Cx3cr1-cre:mTOR^f/f^ mice were reduced, whereas the levels were significantly increased in Cx3cr1-cre: Atg7^f/f^ mice. (Fig S5e & S5f). Likewise, qPCR analysis revealed that the levels of IL-23, IL-1β, and TNFα in purified mononuclear phagocytes were moderately reduced in Cx3cr1-cre:mTOR^f/f^ mice, but significantly elevated in Cx3cr1-cre:Atg7^f/f^ mice (Fig. 4h).

### Rapamycin inhibits induction of proinflammatory cytokines and the fibrotic response independent of T and B cells

**Fig. 5:**
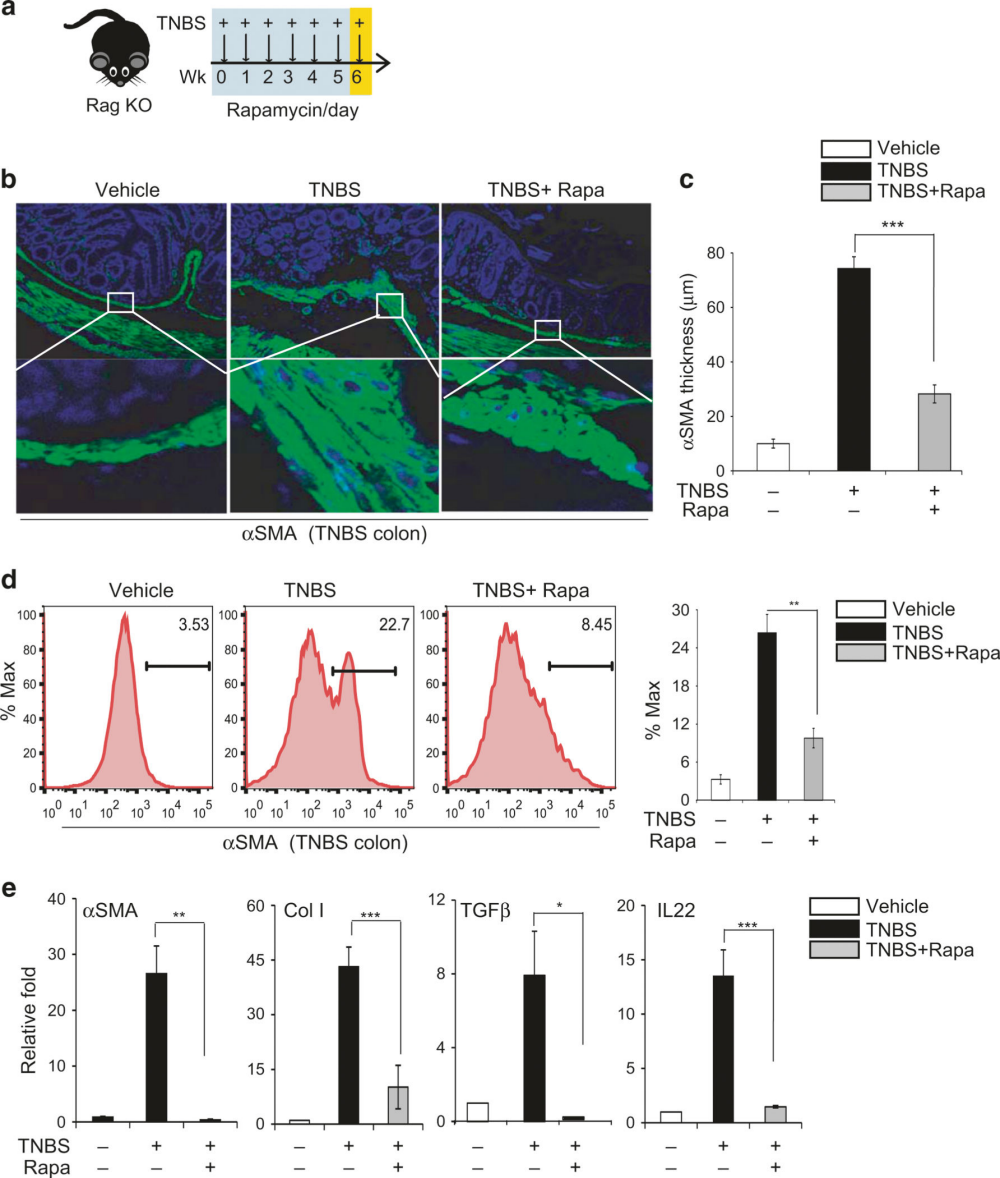
Rapamycin attenuates the TNBS-induced fibrotic response in RAG^−/−^ knockout mice, independent of T and B cells. **a** Diagram showing weekly rectal administration of TNBS and intraperitoneal injection of rapamycin daily (on weekdays) in RAG^−/−^ knockout mice (*n*= 4–7 mice per group); **b** Representative images of αSMA staining; **c** Quantification of the thickness of the submucosal αSMA-positive layer; **d** FACS analysis of αSMA-positive cells in the colon and quantification of αSMA-positive cells; **e** qPCR analysis of fibrosis markers and cytokines in purified CX3Cr1^+^ mononuclear cells from mouse colons. Data are presented as mean ± SEM. *n*= 3, *p < 0.05, ^**^p < 0.01, ^***^p < 0.001

Having demonstrated that pharmacological inhibition of mTOR by rapamycin or genetic inactivation of mTOR in Cx3cr1^+^ mononuclear phagocytes attenuates induction of IL-23 and IL-1β and concurrent intestinal fibrosis, we next examined if the effects we observed require immune cells other than Cx3cr1^+^ mononuclear phagocytes. We employed RAG KO mice, which lack both T and B cells (Fig. 5a). We found that RAG KO mice developed severe intestinal fibrosis in response to TNBS treatment, including shortening of the colon (Fig S6a–S6b) and significant thickening of the αSMA-positive layer of the submucosa (Fig. 5b, c). Moreover, rapamycin treatment remained effective at attenuating both colon shortening (Fig S6a) and thickening of the α-SMA-positive mucosal layer (Fig. 5b, c), and it lowered the population of αSMA-positive cells in FACS staining (Fig. 5d). Finally, there was significant induction of α-SMA, Col-I, TGFβ, and IL-22 in RAG KO mice (Fig. 5e), but again, this induction was attenuated by rapamycin.

### Pro-fibrotic effect of IL-22

**Fig. 6:**
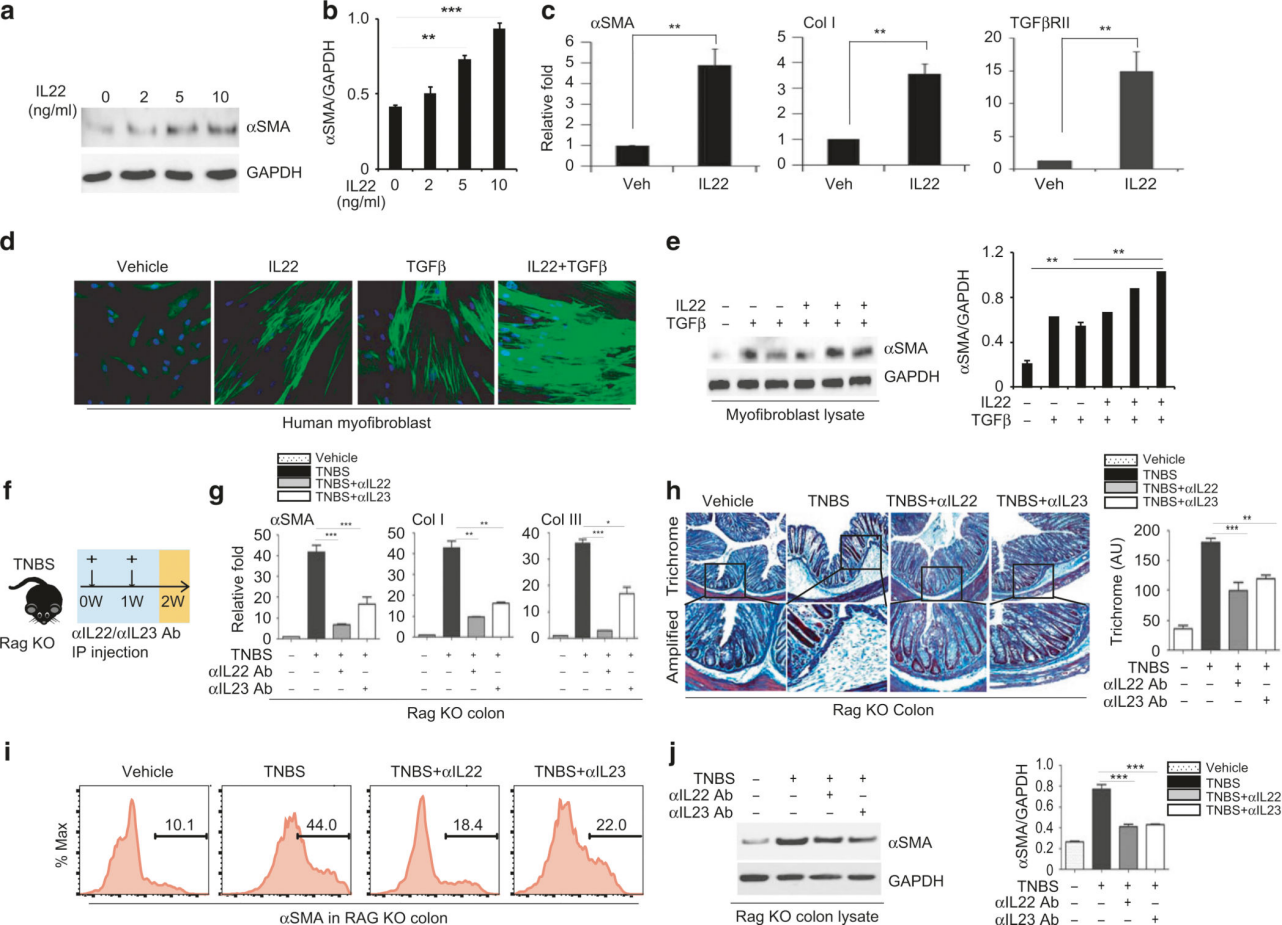
IL-22 promotes proliferation of myofibroblasts. **a** Western blot analysis of αSMA expression in cultured human fibroblasts treated with recombinant IL-22 (0 ng, 2 ng, 5 ng, and 10 ng/ml) for 16 h and **b** quantification of αSMA expression normalized to GAPDH; **c** qPCR analysis of the expression of αSMA, Col-I, and TGFβRII in fibroblasts stimulated with rIL-22 at 10 ng/ml; **d** Representative images of αSMA staining of cultured fibroblasts treated with rIL-22 (10 ng/ml) and TGFβ (10 ng/ml) individually or pre-incubated with rIL-22 (10 ng/ml) for 6 h and then stimulated with TGFβ (10 ng/ml); **e** Western blot analysis of αSMA expression and quantification with normalization to GAPDH control; **f** Schematic diagram representing weekly rectal administration of TNBS and intraperitoneal injection of 50 μl isotype or anti-IL-22 and anti-IL-23 neutralizing antibodies at 100 μg per mouse every other day in wild-type mice; **g** qPCR analysis of the expression of αSMA, Col-I, and Col-III; **h** Representative images of trichrome blue staining for collagen deposition. **i** FACS analysis of αSMA^+^ cells and **j** Western blot analysis of αSMA expression. Data are presented as mean ± SEM. *n*= 3–5 mice/group. ^**^p < 0.01, ^***^p < 0.001

IL-22 regulates tissue repair and remodeling. We established that the level of IL-22 was elevated in fibrotic colon (Figs. 2g and 5e). Accordingly, we determined whether IL-22 plays a role in the fibrotic response. We found that IL-22 stimulated the expression of αSMA in a dose-dependent manner in cultured human fibroblasts, producing an approximately 3-fold increase of αSMA RNA (Fig. 6a, b), and confirmed the proliferation of myofibroblasts. Interestingly, we also observed significant induction of the TGFβ receptor TGFβRII (Fig. 6c). Because the level of TGFβ was elevated in Cx3cr1^+^ mononuclear phagocytes in the colon of TNBS-treated mice, we asked if IL-22 acts synergistically with TGFβ in the fibrotic reaction. Individual treatment with IL-22 and TGFβ increased the expression of αSMA and the proliferation of myofibroblasts (Fig. 6d, e). However, we found that TGFβ was able to elicit a much more robust expression of αSMA and hyper-proliferation of myofibroblasts when the fibroblasts were pre-incubated for 6 h with IL-22. These data suggest that there is a synergistic effect of IL-22 and TGFβ.

More interestingly, in vitro exposure of the mouse lamina propria fraction with IL-23 increased the expression of αSMA (Fig S7a–S7b). This effect on αSMA expression was blocked by anti-IL-22 neutralizing antibody (Fig S7b). To confirm the role of IL-22 in intestinal fibrosis, we performed IL-22 neutralization in vivo.^46–48^ Remarkably, in vivo injection of anti-IL-22 antibody (100 μg/mouse) limited the induction of αSMA and Col-I (Fig S7c–S7e). Histologically, neutralization of IL-22 also attenuated the thickening of the αSMA-positive layer in the TNBS model (Fig S7c–S7d), but had a minimal effect on other cytokines, including IL-23 (Fig S7f). Neutralization of IL-23 reduces induction of IL-22 and IL-17^49–51^ (Fig S7g). Moreover, neutralization of either IL-23 or IL-22 reduced αSMA expression and collagen deposition in Cx3cr1Atg7^f/f^ (Fig S8a–S8f) and RagKO mice (Fig. 6f–j). These data suggest that up-regulation of IL-23/IL-22 axis is critically involved in the fibrotic reaction in the TNBS mouse model. Finally, depletion of CD90 cells^52,53^ attenuates the induction of αSMA, Col-I, and Col-III as well as collagen deposition in RAGKO mice (Fig S9a–9j), suggesting that ILC cells likely contribute to the intestinal fibrosis.

## DISCUSSION

**Fig. 7:**
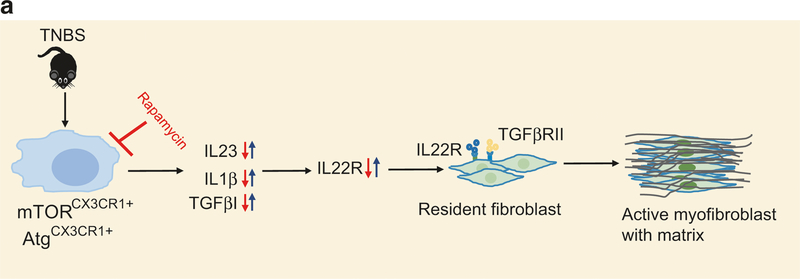
**a** Schematic working model. Intestinal injury induces the expression of IL-23 in Cx3cr1^+^ mononuclear phagocytes in an mTOR/ autophagy-dependent manner. IL-23 stimulates the expression of IL-22. This induction is not necessarily dependent on T and B cells. IL-22 possesses a pro-fibrotic effect by promoting the proliferation and transformation of fibroblasts into myofibroblasts. IL-22 and TGFβ exert a pro-fibrotic effect synergistically. Mechanistically, IL-22 up-regulates the expression of TGFβ RII, thus priming fibroblasts to become fibrotic

Here we revealed that the IL-23/IL-22 axis is up regulated in human Crohn’s patients and in the TNBS model of intestinal fibrosis in both wild-type and Rag^−/−^ mice. This induction of IL-23/IL-22 is associated with activation of the mTOR pathway in intestinal Cx3cr1^+^ mononuclear phagocytes. We further demonstrated that deletion of Atg7 in these phagocytes exacerbates the induction of IL-23/IL-22 in both the intestine and purified mononuclear phagocytes of TNBS-treated mice. Moreover, pharmacological inhibition of mTOR by rapamycin or genetic deletion of mTOR in Cx3cr1^+^ mononuclear phagocytes attenuates the induction of IL-23/IL-22 as well as the resultant fibrosis. In contrast, deletion of Atg7 exacerbates the fibrosis. Finally (Fig. 7), we provided compelling evidence showing that IL-22 promotes the transformation of fibroblasts to myofibroblasts, and neutralization of either IL-23 or IL-22 effectively blocks the fibrosis in TNBS mouse models. Taken together, we revealed a new pro-fibrotic signaling pathway that originates in Cx3cr1^+^ mononuclear phagocytes, in which the mTOR/autophagy pathway up-regulates the IL-23/IL-22 axis to promote fibrosis.

### The mTOR/autophagy pathway in intestinal fibrosis

mTOR signaling was reported to be up-regulated in activated monocytes, macrophages, and dendritic cells.^54,55^ There is abundant evidence showing that this signaling cascade is involved in inflammatory responses; however, its impact on the inflammatory response appears to be bidirectional. Deletion of TSC1, an upstream negative regulator of mTOR, promotes the M1 response to produce TNFα and IL-12p40 in macrophages^56,57^ and attenuates M2 polarization.^57^ Rapamycin is a potent inhibitor of mTORC1.^44,58^ It effectively inhibits the inflammatory response in TSC1KO macrophages^56^ and rescues M2 polarization.^56,57^ However, other studies reported that inhibition of mTOR promotes an inflammatory response. For example, inhibition of mTOR by rapamycin enhances the production of inflammatory cytokines such as TNFα and IL-12p40 (a subunit of IL-23) in response to LPS.^54^ A recent study revealed that deficiency of Lamtor1, a newly identified component of the amino acid-sensing complex in the mTORC1 pathway, enhances inflammatory M1 polarization.^59^ Rapamycin promotes M2 polarization, but fails to reverse M1 polarization.^57^ We found that short-term exposure to rapamycin enhances the induction of IL-23 and IL-1β in LPS-treated macrophages. In contrast, prolonged treatment with rapamycin attenuates the induction of IL-23 and IL-1β while inducing autophagy activity, which is regulated by mTORC1. This finding may explain, in part, why the effect of rapamycin reported in previous studies is inconsistent. Although rapamycin more selectively inhibits mTORC1, it also to some extent inhibits mTORC2 at high doses and prolonged exposure. Interestingly, several recent studies reported that mTORC2 regulates the innate immune responses of macrophages and dendritic cells.^60,61^ In our experimental system, mTOR deletion inactivates both mTORC1 and mTORC2. We therefore cannot entirely rule out the possibility that rapamycin inhibits fibrosis via inhibiting mTORC2. Nevertheless, understanding the cause of the different effects elicited by rapamycin could guide us toward better deployment of this compound in treating inflammatory bowel disease.

There is a considerable body of evidence supporting that the mTOR/autophagy pathway is involved in IBDs and perhaps intestinal fibrosis. Autophagy, residing downstream of mTOR, is a cellular ‘self-eating’ process which is critical for degrading large organelles and unwanted cellular debris, and for mediating host defense against pathogens.^62^ Among the more than 200 genetic loci^25^ which have been identified by GWAS analyses in IBD patients, several genes belong to the mTOR/autophagy pathway, including ATG16L1,^3,5^ NOD2,^6,7^ IRGM,^8,9^ LRRK2^10,11^, and ULK1.^12^ Variants of these genes are associated with a greatly increased risk of IBD,^11,26,27^ and patients with these mutations tend to develop severe intestinal inflammation, stricture, and stenosis.^1,63^ The impaired autophagy appears to exacerbate inflammatory bowel disease via various routes. Autophagy activity is critical in maintaining epithelial barrier function in the intestine. Atg16L1 mutations in Paneth cells lead to abnormalities in the granule exocytosis pathway and to heavy infiltration of monocytes into the muscle layer and mesentery layers.^64^ Mutation of Atg16L1 also compromises auto-phagosome formation and degradation of internalized pathogens^3^ and long-lived proteins.^63^ These defects can lead to bacterial dissemination and severe pathogen-induced inflammation. Autophagy also regulates the inflammatory response in intestinal macrophages and dendritic cells. Deletion of Atg16L1 increases the production of IL-1β and IL-18 in macrophages in response to LPS, and mice lacking Atg16L1 are more susceptible to dextran sulfate sodium-induced colitis.^63^ Atg16L1-deficient dendritic cells fail to induce regulatory T cells to suppress mucosal inflammation.^65^ Pharmacological inhibition of autophagy was reported to up-regulate the expression of IL-23 in cultured macrophages,^66^ but it remains unclear if such regulation has any role in intestinal fibrosis. Autophagy activity is expressed in nearly all types of intestinal cells and clearly implicated in intestinal homeostasis and inflammation. Here we revealed that autophagy in Cx3cr1^+^ mononuclear phagocytes contributes significantly to intestinal fibrosis.

### Defining the role of the IL-23/IL-22 axis in intestinal fibrosis mediated by Cx3cr1 mononuclear phagocytes

In the intestinal lamina propria, several types of mononuclear phagocytes express Cx3cr1 receptors, including macrophages and subsets of dendritic cells. These cells mediate mucosal immunity and gut homeostasis. Both macrophages and dendritic cells can produce IL-23.^22,34^ IL-23/IL-23R-mediated IL-22 pathways gained significant attention in recent years owing to their central importance in antimicrobial immunity, inflammation, and tissue repair.^37,67^ IL-23 acts as a pleiotropic cytokine secreted from activated macrophages and dendritic cells, and has been shown to induce secretion of both IL-17 and IL-22 from T cells^18^ and ILCs.^22,67^ The levels of IL-23 and IL-22 are elevated in the intestine of patients with CD.^32,68^ Genome-wide association studies revealed an association of the IL-23R gene with CD.^11,26,27^_Cx3cr1_^+^ mononuclear phagocytes are critical in supporting the production of IL-22 from ILC3 cells.^22^ Mice with depletion of this population of cells displayed impaired induction of IL-22 and were more susceptible to bacteria-induced severe colitis, thereby confirming a critical role of the IL-23/IL-22 axis in intestinal homeostasis and mucosal immune defense. Mice deficient for IL-23p19 are more susceptible to colitis in the experimental T cell-mediated TNBS model.^28^ However, the IL-23/IL-22 axis was also reported to exacerbate inflammation in a chronic/adaptive colitis model.^32^ These observations highlight the complex role that this axis plays in intestinal pathogenesis.

Both T cells and ILCs are implicated in IL-22 secretion in the intestine.^18,68^ Recently, ILC3 cells have been implicated in the clearance of pathogens and tissue repair.^69–71^ Their role in intestinal fibrosis needs to be further investigated in future studies. We found that induction of IL-23 and IL-22 and progression of intestinal fibrosis continued to occur in the Rag^−/−^ KO mouse model which lacks both T and B cells. These data suggest that, other than T cells, ILCs, and most likely ILC3 cells, may be an important alternative source of IL-22.^22^ Our data from the CD90 depletion experiment strongly suggest that ILCs are involved in the fibrotic response.

Intestinal fibrosis could be thought of as an over-reactive repair process responding to inflammation and injury. IL-22 promotes intestinal epithelial regeneration and wound healing.^37^ A recent study reported that IL-22 promotes fibrogenesis in acute skin wounding,^23^ but does the opposite in lung fibrosis.^38^ In the present study, our data revealed that stimulation by recombinant IL-22 promotes the transformation of fibroblasts into myofibroblasts. Neutralization of IL-22 attenuates the fibrotic reaction. These data confirm that IL-22 possesses a pro-fibrotic effect in the intestine. This finding extends the known set of functions of IL-22, including intestinal homeostasis and repair.^21,72,73^ Finally, among the array of cytokines, chemokines, and inflammatory mediators, TGFβ is well known to be involved in intestinal fibrosis. Our study revealed a novel myofibroblast-promoting activity of IL-22, which extends the repertoires of pro-fibrotic factors. We also found that IL-22 and TGFβ act synergistically to promote the fibrotic response in cultured fibroblasts. A next step would be to understand how these pro-fibrotic factors interact to bring about intestinal fibrosis.

Intestinal fibrosis is a consequence of inflammation. CD and ulcerative colitis (UC) are both forms of IBD and have overlapping genetic profiles. However, the severity of fibrosis does not always positively correlate with inflammatory status, perhaps due to different adaptive immune responses.^32,74^ In the present study, we found that, in the absence of major immune cells (in Rag^−/−^ KO mice), the intestine still developed a significant fibrotic response, suggesting a critical role of low-grade inflammation in fibrosis. Indeed, prevention of low-grade inflammation has proven to be beneficial in CD clinical trials.^75,76^ It has helped in moving away from steroid therapies for CD, and has reduced the need for surgery. In light of a pathological role of low-grade inflammation in many chronic diseases, including CD, it is conceivable that the elevated activity of the IL-23/IL-22 axis driven by the innate immune system, including Cx3cr1^+^ mononuclear phagocytes and ILCs, could be the pathological lynchpin for the excessive fibrotic reaction in intestine experiencing chronic injury or disruption of tissue homeostasis.

In summary, mTOR/autophagy has been implicated in IBD pathogenesis and severe fibrosis. While the mTOR/autophagy process occurs in nearly all types of intestinal and immune cells and is implicated in various aspects of intestinal homeostasis, we pinpointed the critical role of aberrant mTOR/autophagy signaling in Cx3cr1^+^ mononuclear phagocytes that drives their pathologic pro-fibrotic activity via (at least in part) the IL-23/IL-22 axis. Therefore, the IL-23/IL-22 axis represents a viable target for the prevention of intestinal fibrosis. The challenge we now face is how to effectively target the pro-fibrotic component of IL-22 while preserving its other biological activities critical to maintaining intestinal homeostasis and promoting tissue repair.

## MATERIALS AND METHODS

### Animals

C57BL/6 wild-type (Stock #000664, Jackson laboratory), Rag^−/−^ s(Rag1tm1Mom/J, Stock #002216, Jackson laboratory), and mTOR-flox/flox (B6.129S4-Mtortm1.2Koz/mTOR) mice were procured from Jackson Laboratory (Bar Harbor, ME). Cx3cr1-Cre mice were obtained from the Mutant Mouse Resource and Research Center (Stock#16959 MMRRC). Dr. Masaaki Komatsu donated the Atg7^flox/flox^ mice. Atg7^f/f^ and mTOR^f/f^ mice were further crossed with CX3Cr1-Cre mice to generate CX3Cr1-Cre: Atg7^f/f^ and CX3Cr1: mTOR^f/f^. All mice were either already on a C57/BL6 background or backcrossed with C57/BL6 wild-type mice for more than 10 generations. All animals were housed and bred in a specific pathogen-free unit, with a 12-h light cycle (lights on at 7:00 A.M.), temperature- and humidity-controlled, at Albany Medical Center’s animal facility. Efforts made to minimize suffering and unnecessary use of animals, according to the guidelines set by the Institutional Animal Care and Use Committee (IACUC) and the National Institutes of Health Guide for the Care and Use of Laboratory Animals.

### Human intestinal specimens

All intestinal tissues (ileocolonic) were obtained from Albany Medical Center (Albany NY). Intestinal surgical specimens listed in Table 1 were obtained from three female patients, ranging from 24 to 40 years old. CD was diagnosed in these patients after surgery. Control ileocolonic tissues were obtained from surgical resection from 3 patients (1 male and 2 females) who had no history of IBDs. Intestinal slices were prepared from paraffin-embedded tissues sectioned at 5 μm and stained with αSMA or trichrome blue. In addition, fresh ileum biopsies listed in Table 2 were acquired from IBD clinics at Albany Medical Center (Albany, NY). They were processed for histological analysis of collagen deposition via trichrome blue staining or αSMA staining of myofibroblasts. Total RNAs and protein lysates were also prepared for western blot analysis for the expression of αSMA and qPCR analysis of fibrosis markers and cytokines. All human tissues were obtained in accordance with a protocol approved by the Albany Medical College Institute Review Board and Committee on Human Research.

### Induction of TNBS fibrosis and rapamycin treatment

The TNBS (2,4,6-trinitrobenzene sulfonic acid) (Cat#92823, Sigma) mouse model was established according to previously published protocols.^77^ Briefly, adult mice were shaved on the neck area and presensitized to TNBS via dermal exposure. Colitis was then induced 8 days later by using intra-rectal administration weekly for 6 weeks. Four mg TNBS (in 25% ethanol) was applied in a 100-μl enema using a 1-ml syringe attached to a 3.5 French polyurethane catheter; the control mice received 100 μl of 25% ethanol. Mice were anesthetized with pentobarbital 25 mg/kg i.p. or exposed to isoflurane during TNBS administration. The large intestine was removed for all assays. Control and TNBS-treated mice were treated with rapamycin (Cat#1003799, LC Laboratories) at 2 mg/kg/day or vehicle (5% Tween-20 and 4% ethanol) via intraperitoneal injection every weekday for 3–6 weeks.

### Immuno-histopathologic assessment of gut fibrosis

All mouse and human tissues were fixed in 4% paraformaldehyde for 48 h and then in 70% ethanol for 16 h. The fixed tissues were then embedded in paraffin, sliced in the Histology Core at Albany Medical Center and stained with H&E (Cat#HXMMHPT, American Mastertech Kit) and Trichrome Blue (Cat#STOSTBPT, American Mastertech Kit) according to manufacturer’s instructions to detect collagen.^78^ For staining of αSMA, sections were incubated with mouse monoclonal antibody specific for αSMA (Cat#149760–80, Clone#M1/77, Biolegend, USA), along with goat anti-mouse IgG (H + L) conjugated with Alexa Fluor 488 (Cat#11029, Life Technologies, USA) as a secondary antibody. In brief, colon sections were incubated in blocking buffer (0.2% Triton X-100 and 5% normal goat serum in 1X PBS) for 1 h at room temperature, and then incubated in primary antibody solution (0.2% Triton X-100 and 3% normal goat serum in 1X PBS; anti-αSMA 1:200) at 4 °C overnight, followed by washing for 20 min 3 times. The sections were then incubated in buffer containing fluorophore-conjugated secondary antibodies at room temperature for 1 h. Nuclei were counterstained with DAPI and coverslips were applied with Fluor mount G (Cat# 0100–01, Southern Biotech), and sealed with nail polish. All images were acquired using a Zeiss LSM 880 confocal microscope and processed with Zen black 2.1 or Zen blue lite 2.3 (Carl Zeiss). Immunofluorescence or trichrome blue staining signals were quantified using an average of multiple selected areas in the same section by using the NIH image analysis software Image-J.

### Isolation of intestinal lamia propria and purification of mononuclear phagocytes

In brief, colons were longitudinally opened in ice-cold Hank’s Balanced Salt Solution (HBSS) (Cat#21020-CV, Corning) and washed 3–4 times. Colon tissues were then cut into 1-cm pieces and incubated in 5 ml of pre-digestion solution (1X HBSS containing 5% FBS, 1 mM DTT, and 5 mM EDTA) in a 50-ml tube rotated at 100 rpm for 20 min at 37 °C in an incubator. The detached colonic epithelial cells were discarded by passing them through a 40-μm cell strainer. The remaining tissues were washed and further digested in buffer containing Collagenase type IV (Cat# 1088866001, Roche), and DNase I (Cat# D263–5vl, Roche), (1X HBSS containing 5% FBS, 1 mM DTT, and 5 mM EDTA + Collagenase + Dnase I) at 37 °C for 20 min under slow rotation. After incubation, the digestions were vortexed vigorously for 20 s. The cell suspensions were passed through a 40-μm cell strainer. Single-cell suspensions were pelleted and the cells were resuspended in 10 ml of 30% Percoll solution (Cat# 17–0891-01, GE Healthcare), and overlaid on top of 5 ml of 70% Percoll solution in a 15-ml tube. The 30/70 Percoll gradients were centrifuged without braking at 1000×*g* at room temperature. The white ring phase contained lamina propria lymphocytes, and these were collected and resuspended in ice-cold HBSS and centrifuged at 500×*g* at 20 °C for 10 min. The pellets were resuspended in FACS buffer. Derived single-cell suspensions were either analyzed using an LSR II Flow-Cytometer (BD Biosciences) or used for purification of mononuclear phagocytes with PE magnetic beads. For FACS analysis, cells were first blocked with anti-mouse CD16/CD32 Fc block (Cat#14–9760-80, Biolegend) prior to staining with antibodies against surface or intracellular markers. To purify mononuclear monocytes, cell suspensions were blocked with anti-mouse CD16/CD32 Fc block, followed by anti-Cx3cr1-PE (Cat# 149006, Biolegend) along with anti-PE Microbeads (Cat#130–105639, Miltenyi) to capture bound cells.

### RNA isolation, RT-PCR, real-time PCR

Total RNA was extracted from colon tissue and single-cell culture according to the manufacturer’s instructions, using TRIzol Reagent (Cat#: 15596018, Life Technologies). To isolate RNA from colon, colon tissues were excised, rinsed with PBS and then briefly homogenized in TRIzol Reagent. RNase-free glycogen of 0.5 μl was added from a 20-μg/ml stock solution (Roche, Cat#10901393001) to improve the recovery of total RNA prior to RNA precipitation with isopropanol. RNA pellets were resuspended in 50 μl of RNase-free water (Cat#BP561–1 Fisher Scientific) and incubated at 55 °C for 10 min. RNA concentrations were determined by using a Smart-Spec plus spectrophotometer (Bio-Rad). cDNA was synthesized from 0.2–1 μg of total RNA via reverse transcription using a Verso cDNA Synthesis Kit (Cat#: AB-1453/B, ThermoScientific) in a total volume of 20 μl. The cDNA templates were further diluted 2–3 times in water. Two microliters of diluted templates were used for real-time PCR. RT-PCR was performed in a 96-well PCR plate (Cat#MLL9601, Bio-Rad) using a SYBR Green qPCR Master Mix kit (Cat#: A25777, Applied Biosystems) in a Step One Plus Real-time PCR System (Applied Biosystems). Each sample was evaluated in triplicate. The CT value was used to calculate the fold change of RNA abundance after normalization to GAPDH. All primer sequences are provided in supplementary Table 1.

### Western blotting

The vendors and catalog numbers for the primary antibodies are as follows: Anti-GAPDH (Cat # 5174, Clone# D16H11, Cell Signaling), Phospho-S6 Ribosomal Protein (Ser235/236) (Cat # 4858, Clone# D57.2.2E, Cell Signaling), S6 Ribosomal Protein (Cat # 2217, Cell Signaling), Phospho-p70 S6 Kinase (Clone#S371, Cell Signaling), p70 S6 Kinase (Cat # 2708, Clone#49D7, Cell Signaling), αSMA (Cat#149760–80, Clone#M1/77, Cell Signaling). Western blotting was performed as previously described.^79,78^ Briefly, cells were lysed in RIPA buffer (1% NP40) and then resolved in an 8% Bis-Tris gel at a constant voltage of 80 V. The protein was transferred to nitrocellulose membranes in cold transfer buffer for 2 h at 4 °C, followed by blocked in 5% nonfat dry milk in TBST (25 mM Tris–HCl, pH 7.4, 1.5 M NaCl, 0.05% Tween-20) for 1 h at room temperature and then incubated in primary antibodies at 4 °C overnight. Membranes were washed 3–4 times at 15 min intervals and incubated with HRP-conjugated secondary antibody (Sigma) (1:10,000) in 5% milk in TBST and detected using an ECL (Pierce) developing kit. The intensities of signals were normalized to GAPDH as a loading control for densitometry analysis.

### Flow cytometry analysis

For FACS analysis, we modified our previous protocol^79,80^ as follows: cells were resuspended in 50 ml of FACS buffer (1% BSA, 2 mM EDTA and 0.1% sodium azide in PBS, pH 7.4) (5–10 × 10^5^ cells/tube) and incubated on ice for 10 min with anti-mouse CD16/CD32 (Cat#14–9760-80, Biolegend) at 1:50 dilution to block Fc receptors. Cells washed with 500 ml of ice-cold FACS buffer twice to remove unbound anti-CD16/CD32 prior to cell-surface or intracellular staining. To evaluate colon lamina propria, colonic single-cell suspensions (10^6^ cells/50 ml) were stained with anti-CD11b (Cat#101226, Clone#M1/70, Biolegend), anti-CD40 (Cat#101226, Biolegend), anti-CD80 (Cat#104717, Clone#16–10A1, Biolegend), anti-CD86 (Cat# 25–0862-80, Clone#GL1, Biolegend), anti-MHC-II (Cat# 107621, Clone#M5/114.15.2, Biolegend) at 1:100 dilution on ice for 30 min to label surface markers. Stained cells then washed with 500 μl of ice-cold FACS buffer twice to remove unbound antibodies and analyzed immediately by LSR II Flow Cytometer (BD Biosciences>). For detecting intracellular cytokines, cell suspensions were incubated for 3 h with PMA (50 ng/mL) (Cat#P8139, Sigma) and Ionomycin (1 μg/mL) in the presence of BD Golgi Plug (Cat# 555028, BD Bioscience), followed by incubation on ice for 10 min with anti-mouse CD16/CD32 at 1:50 dilution to block Fc receptors. Cells were washed with 500 ml of ice-cold FACS buffer twice to remove unbound anti-CD16/CD32 prior to cell surface staining with anti-CD11b (Biolegend) and anti-F4/80 (Biolegend) at 1:1000 dilution on ice for 30 min. These surface-stained cells were then fixed and permeabilized using a Fixation/Permeabilization Solution Kit (Cat# 555028, BD Bioscience) for 20 min according to the manufacturer’s instructions. Cells were then incubated for intracellular staining with antiIL-23 (Cat# 53–7023-80, Clone#fc23cpg, Biolegend) and anti-IL-1β, (Cat# IC4013P, Clone#166931, R&D) on ice for 30 min. Cells were washed in FACS buffer twice on ice for 5 min and immediately subjected to FACS analysis. To determine αSMA level, single cells from colons were fixed and permeabilized using a Fixation/Permeabilization Solution Kit (Cat# 555028, BD Bioscience), according to the manufacturer’s instructions. Cells were then stained with anti-αSMA-AF488 (Cat#53–9760-82, Clone#1A4, Biolegend) at 1:1000 dilution on ice for 30 min. Cells were washed in FACS buffer twice on ice for 5 min and immediately subjected to FACS analysis. Finally, the cells were analyzed using a FACS LSRII (BD Bioscience) and data were analyzed using Flow Jo software.

For isolation of P3 and P4 fraction of Cx3cr1^+^ cells, colon lamina propria fractions were prepared from control and TNBS treated-mice and stained for CD64 (Cat# 139315, Clone#X54–5/7.1, Biolegend), CD11c (Cat# 17–0114-81, Clone#N41B, Invitrogen), CD11b(Cat# 101226, Clone#M1/70, Biolegend), CX3CR1(Cat# 149006, Clone#SA011F11, Biolegend), Ly6C (Cat# 128013, Clone#HK1.4, Biolegend), and MHCII (Cat# 107622, Clone#M5/114, Biolegend). P3 + P4 (CD11c-CD64 + CD11b + CX3Cr1 + Ly6C-) cell populations were acquired by FACS sorted using FACS ARIA (BD Bioscience). Sorted cells were lyzed and total RNAs were prepared for detection of cytokines.

### Cytokine neutralization and cell depletion

Mice were administrated with anti-IL-22 antibody (Clone#IL22JOP, Cat#16–7222-85 eBioscience), anti-IL-23 antibody (Clone#G23–8, Cat#16–7232-85, eBioscience), and isotype control via i.p. injection at 100 μg/mouse every other day for 2 weeks. For the ILC depletion experiments, anti-CD90.2 (Clone# 30H12 Cat# BE0066 from Bioxcel, West Lebanon, NH) or isotype were injected intraperitoneally 300 μg/mouse every other day for 2 weeks.

### Statistical analysis

Data were analyzed with appropriate tests using Graphpad Prism 7, GraphPad Software Inc. (San Diego, CA) for comparisons between two groups or wild-type and the KO animals. Data are shown as the mean ± SEM. In all analyses, *P* < 0.05 was considered significant (*P < 0.05; ^**^P < 0.01; ^***^P < 0.001). Student’s *t*-test was used to test the differences between two groups and ANOVA test was used for analysis of more than two groups.

## Supplementary Material

supplemental file
