# Supplementary material for: Induction of autophagy in Cx3cr1+ mononuclear cells limits IL-23/IL-22 axis-mediated intestinal fibrosis
Source: Mucosal Immunol. Author manuscript; Available in PMC 2019 Dec 23. (PMC6927046; doi:10.1038/s41385-019-0146-4)
Supplement: supplemental file [file NIHMS1060780-supplement-supplemental_file.pdf]

Table 1

**Surgical patients**			
**Age (year)**	24	40	29
**gender**	female	female	female
**smoking**	none	none	none
**Duration fo disease**	2 months	1 months	o months
**CD location**			
Ileal			
(L1)			
Colonic (L2)			
Ileocolonic (L3)	+	+	+
Upper GI(L4a)			
Upper GI(L4b)			
**Types of CD**			
Inflammatory (B1)	+	+	+
Stricturing (B2)			
Penetrating (B3)	+	+	+
**Types of UC**			
Proctitis (E1)			
left sided colitis (E2)			
Extensive (E3)			
**Medication**			
Immunosuppressants			
Biologics			
Antibiotics	+		+
Bedesonide			
Systemic steroid		+	
5-aminosalicylic acids			
**Disease activity metrics**			
**laboratory data**			
White blood cells	6.3	9.4	10.7
Hematochrit	36.8	27.6	26.5
Platelet counts	551	584	481
Erythrocyte sedimentation rate	60	35.2	
C-reactive protein	44.5	44.5	
**Endoscopy scores**			
CD-Rugeerts score	i3	i4	i4
UC-Mayo score			
**Pathology**			
Ulceration	+	+	+
Mural scarring	+		
Fissure/Fistula	+	+	+
Abscess			+
Granulomas & Giant cells	+	+	+

Table 2

	CD remission	Active CD	CD remission	Active CD	Active CD	UC	UC	CD remission	Active CD	CD remission	CD remission	UC
**Patients**	Patient 1	Patient 2	Patient 3	patient 4	patient 5	patient 6	patient 7	patient 8	patient 9	patient 10	patient 11	patient 12
Age	36	48	54	36	28	39	30	43	39	29	37	29
gender	female	male	female	female	female	female	female	female	female	male	feamle	male
smoking	none	remote	remote 10y	active 1ppd	none		none	none	none	none	none	none
**Duration fo disease (years)**	3	19	12	1	22	16	2	13	26	2	6	7
**CD location**												
Ileal (L1)	+	+									+	
Colonic (L2)			+									
Ileocolonic (L3)				+	+			+	+	+		
Upper GI(L4a)												
Upper GI(L4b)				+					+			
**Types of CD**												
inflammatory (B1)	+	+		+	+			+		+	+	
Stricturing (B2)									+			
penetrating (B3) (Fistula or abscess)			+						+		+	
**Types of UC**												
Proctitis (E1)						E1						E1
Left sided colitis (E2)												
Extensive (E3)							E3					
**Hisotry of IBD-related surgery**		+							+		+	
**Medication**												
Immunosuppressants		+		+					+			
Duration of Biologics (years)	3.5	1	5.5	0.6	4.5			8				
Bedesonide (year)				1						1		
Systemic steroid (year)							1					
5-aminosalicylic acids						1	1				4.8	
**Disease activity metrics**												
**Laboratory data**												
WBC	14.2	3.2	4.9	13.2	9.4	7.5	14.5	11.9	8.5	3.8	6.2	7.8
Hematochrit	40.9	39	40	38.9	39.6	39.4	39.9	35.3	43	46	42.7	44.4
Platelet counts	206	249	246	319	274	407	319	387	295	184	245	253
Erythrocyte sedimentation rate	8	6	9	20	9	13	22	76	7	3	5	6
C-reactive protein	0.2	1.6	2.9	12.6	0.5	1.3	66.4	9.7	3.8	0.6	0.3	0.9
**Endoscopy scores**												
CD-Rugeerts score	i0	i1-i2	i0	i3	i0			i0			i0	
UC-Mayo score						0	4					1
**Pathology**												
Ileitis		+		+	+		+					
Colitis												
Proctitis						+						+
Colon mucosal architecture distortion									+			

Supplemntary Table 3

**Oligonucleotides**	**Sequence**	**Source**	**Identifier**
Genename			
mouse Gapdh Fwd	TTCACCACCATGGAGAAGGC	Life Technology	N/A
mouse Gapdh Rev	GGCATGGACTGTGGTCATGA	Life Technology	N/A
mouse IL1β Fwd	GTTCAGGACAGCGACTTCTGGA	Life Technology	N/A
mouse IL1β Rev	GAAGGAGAACTCCGCTGACTCT	Life Technology	N/A
mouse IL23 Fwd	GCCCAGATGGTTTTTGGGTTC	Life Technology	N/A
mouse IL23 Rev	GCAAGGTACGGATGATAATGAGG	Life Technology	N/A
mouse IL22 Fwd	ACCCTCCCACTTCCTGCTGTTT	Life Technology	N/A
mouse IL22 Rev	CTGTCTGCCTCTTTTGGTCAGG	Life Technology	N/A
mouse IL17A Fwd	CCATGCTTGACTTGGACGATGAC	Life Technology	N/A
mouse IL17A Rev	TGGCGATGTGACTGGTGAGTTC	Life Technology	N/A
mouse IL17F Fwd	GTGGAAGACAGTATCTGCTGCC	Life Technology	N/A
mouse IL17F Rev	AGGCTTGCGGCAGGATTTTGAG	Life Technology	N/A
mouse αSMA Fwd	ACTACCAAGCCAGTGCTGCGAA	Life Technology	N/A
mouse αSMA Rev	ATCACAGGCGAAGTCCAATCCG	Life Technology	N/A
mouse Colagen-I Fwd	CAGGAGAAGACCCAGCAAGTCA	Life Technology	N/A
mouse Colagen-I Rev	CTCACAGCTCTAGTCCTCTTGG	Life Technology	N/A
mouse Colagen-III Fwd	TGGGCACCATCTTCATCATTC	Life Technology	N/A
mouse Colagen-III Rev	GGTCACCCAGCACACCACTT	Life Technology	N/A
mouse TGFβ Fwd	ATCAACGCTACACTGCATCTTGGCTT	Life Technology	N/A
mouse TGFβ Rev	CCTCAAACTTGGCTACTCATGAATGC	Life Technology	N/A
mouseTGFβRII Fwd	CCCCTGGAGACAATACCAGC	Life Technology	N/A
mouseTGFβRII Rev	TTAGCCAACCACCACACAATG	Life Technology	N/A
mouse CTGF Fwd	TGAGCAGGATGGAGAATTACAGG	Life Technology	N/A
mouse CTGF Rev	GTCCAAGTTCATCTTCTAGGCAC	Life Technology	N/A
mGMCSF Fwd	ATGCCTGTCACGTTGAATGAAG	Life Technology	N/A
mGMCSF Rev	GCGGGTCTGCACACATGTTA	Life Technology	N/A
mIL8 Fwd	GTG CAG TTT TGC CAA GGA GT	Life Technology	N/A
mIL8 Rev	TTATGAATTCTCAGCCCTCTTCAAAAACTTCTC	Life Technology	N/A
mIFNβ Fwd	GCCTCGTGCTGTCGGACC	Life Technology	N/A
mIFNβ Rev	TGTCGTTGCTTGGTTCTCCTTG	Life Technology	N/A
mIFNα Fwd	CCTGAGAA/GAGAAGAAACACAGCC	Life Technology	N/A
mIFNα Rev	GGCTCTCCAGAC/TTTCTGCTCTG	Life Technology	N/A
			N/A
hActin_Fwd	CTCTTCCAGCCTTCCTTCCT	Life Technology	N/A
hActin_Rev	AGCACTGTGTTGGCGTACAG	Life Technology	N/A
hIL4_Fwd	CCAACTGCTTCCCCCTCTG	Life Technology	N/A
hIL4_Rev	TCTGTTACGGTCAACTCGGTG	Life Technology	N/A
hIL5_Fwd	GCTTCTGCATTTGAGTTTGCTAGCT	Life Technology	N/A
hIL5_Rev	TGGCCGTCAATGTATTTCTTTATTAAG	Life Technology	N/A
hIL13_Fwd	AGCCCTGGATTCCCTGAC	Life Technology	N/A
hIL13_Rev	GCTGAGACCCTGAGCACTAG	Life Technology	N/A
hGMCSF_Fwd	CACTGCTGCTGAGATGAATGAAA	Life Technology	N/A
hGMCSF_Rev	GTCTGTAGGCAGGTCGGCTC	Life Technology	N/A
hIL1β_Fwd	ACAGATGAAGTGCTCCTTCCA	Life Technology	N/A
hIL1β_Rev	GTCGGAGATTCGTAGCTGGAT	Life Technology	N/A
hIL8_Fwd	ATGACTTCCAAGCTGGCCGTGGCT	Life Technology	N/A
hIL8_Rev	TCTCAGCCCTCTTCAAAAACTTCTC	Life Technology	N/A
hIL23_Fwd	CGTCTCCTTCTCCGCTTCAA	Life Technology	N/A
hIL23_Rev	ACCCGGGCGGCTACAG	Life Technology	N/A
haSMA_Fwd	TGACCCAGATTATGTTTGA	Life Technology	N/A
haSMA_Rev	GCTGTTATAGGTGGTTTCG	Life Technology	N/A
hCollagen_Fwd	CCTGGCAAAGACGGACTCAAC	Life Technology	N/A
hCollagen_Rev	GCTGAAGTCATAACCGCCACTG	Life Technology	N/A
hIFNα_Fwd	GACTCCATCTTGGCTGTGA	Life Technology	N/A
hIFNα_Rev	TGATTTCTGCTCTGACAACCT	Life Technology	N/A
hIFNβ_Fwd	CTCCTGTTGTGCTTCTCCACT	Life Technology	N/A
hIFNβ_Rev	GGCAGTATTCAAGCCTCCCA	Life Technology	N/A

Supplementary Table 4
Reagent and resources

Experimental Models: Organisms/Strains	Source	Identifier
Mouse: C57BL/6J	Jackson Laboratories	664
Mouse: Rag1tm1Mom/J Jackson laboratory Stock #002216	Jackson Laboratories	2216
Mouse: CX3Cr1-CRE	Jackson Laboratories	16959
Mouse: mTORf/f	Jackson Laboratories	651
Mouse: Atg7f/f	Dr. Masaaki Komatsu	N/A

Experimental Models: Cell Lines	Source	Identifier
Human Fibroblast cell	Dr Paul	N/A

Antibody	Source	Identifier
Anti-mouse purified CD16/32 Fc block	Biolegend Inc	14-9760-80
Anti-mouse CD11b, APCCy7 conjugated, Clone#M1/70	Biolegend Inc	101226
Anti-mouse F4/80, PE conjugated, Clone#BM8	Biolegend Inc	12480182
Anti-mouse CD40, APCCy7 conjugated, Clone#Mar32	Biolegend Inc	124625
Anti-mouse CD80, Alexa Fluor® 647 conjugated, Clone#16-10A1	Biolegend Inc	104717
Anti-mouse CD86, PE-Cyanine7 conjugated, Clone#GL1	Biolegend Inc	25-0862-80
Anti-mouse I-A/I-E, Alexafluor700 conjugated, Clone#M5/114.15.2	Biolegend Inc	107621
Anti-mouse CX3cr1 PE conjugated, Clone#SA011F11	Biolegend	149006
Anti-mouse IL23, PE conjugated, Clone#fc23cpg	Biolegend Inc	53-7023-80
Anti-mouse IL1β, PE conjugated, Clone#166931	R&D	IC4013P
Anti-mouse αSMA, purified Clone#M1/77	e-bioscience	149760-80
Anti-mouse αSMA, AF488 conjugated, Clone#1A4	e-bioscience	53-9760-82
Anti mouse CD64, FITC conjugated, Clone#X54-5/7.1	Biolegend Inc	139315
Anti mouse CD11b, APCCy7 conjugated, Clone#M1/70	Biolegend Inc	101226
Anti mouse CX3Cr1, PE conjugated, Clone#SA011F11	Biolegend Inc	149006
Anti mouse Ly6C, Pacififc Blue conjugated, Clone#HK1.4	Biolegend Inc	128013
Anti mouse MHC II, Alexafluor 700 conjugated, Clone#M5/114	Biolegend Inc	107622
Anti mouse CD11c, APC conjugated, Clone#N41B	Invitrogen	17-0114-81
Anti mouse CD90, PECy7 conjugated, Clone#OX-7	Biolegend Inc	202518
Ant-mouse IL22 neutralzing antibody, Clone#IL22JOP	e-bioscience	16-7222-85
Ant-mouse IL23 neutralzing antibody, Clone#G23-8	e-bioscience	16-7232-81
Ant-mouse CD90 depeltion antibody, Clone#30H12	Bioexcell	BE0212
Rabbit Anti mouse Phospho-S6 Ribosomal Protein (Ser235/236), Clone# D57.2.2E	Cell signaling	4858
Rabbit Anti mouse S6 Ribosomal Protein	Cell signaling	2217
Rabbit Anti mouse Phospho-p70 S6 Kinase, Clone#S371	Cell signaling	9208
Rabbit Anti mouse p70 S6 Kinase, Clone#49D7	Cell signaling	2708
Rabbit Anti mouse LC3BI/BII	Cell signaling	4108
Rabbit Anti mouse p62	Cell signaling	5114
Rabbit Anti mouse GAPDH, Clone# D16H11	Cell signaling	5174
Goat anti-Rabbit IgG (H+L) Secondary Antibody, Alexa Fluor 488 conjugate	e-bioscience	5018
Goat anti-mouse IgG (H+L) Secondary Antibody, Alexa Fluor 488 conjugate	e-bioscience	
Anti-rabbit IgG, HRP-linked Antibody	e-bioscience	7074

Chemicals, Peptides, and Recombinant Proteins+C88AA44:C83	Source	Identifier
TNBS	Sigma	92823
Rapamycin	LC LABORATORIES	1003799
recombinant mouse IL23	Biolegend Inc	1000871
recombinant IL-1 beta	Biolegend Inc	IC4013P
recombinant mouse IL22	Biolegend Inc	
recombinant mouse TGFb1	Biolegend Inc	
Collagenase type IV	Roche	1088866001
DNase I from bovine pancreas	Roche	D263-5vl
PMA/Ionomycin salt	Sigma	P8139
DMEM	Corning	10013-CV
Mouse M-CSF	Shennandoah	200-08
Percol	GE Healthcare	17-0891-01
Bovine Serum Albumin	InvivoGen	tlrl-isdn
TRIzol	Life technologies	15596018
Glycogen	Roche	10901393001

Critical Commercial Assays	Source	Identifier
Verso cDNA Synthesis Kit	Thermofisher	AB1453B
PowerUp™ SYBR™ Green Master Mix	Thermofisher	A25777
Fixation/Permeabilization Solution Kit with BD GolgiPlug kit	BD Bioscience	555028
Trichorme staining kit	American Mastertech Kit	STOSTBPT
H&E kit	American Mastertech Kit	HXMMHPT
IL22 ELISA Kit	e-bioscience	88-7422-22
IL23 ELISA Kit	Invitrogen	88-7230-22
IL1β ELISA Kit	Invitrogen	88-7013-22

Other materials	Source	Identifier
Anti-PE MicroBeads	Miltenyi	130-105-639
MS Columns	Miltenyi	130-042-201
Falcon® 40µm Cell Strainer	Corning	352340

Softwares	Source	Identifier
FlowJo	FlowJo LLC	www.flowjo.com
Graphpad Prism 7	GraphPad Software Inc	www.graphpad.com
Zen black 2.1	Carl Zeiss	www.zeis.com
Zen blue lite 2.3	Carl Zeiss	www.zeis.com
Image J	NIH	www.imagej.nih.gov/ij/

Supplementary figure legends

Supplementary Figure 1

(a) Mouse body weight over the course of weekly TNBS treatment; **(b)** Western blot analysis of αSMA expression in the lysates prepared from mouse colons treated with TNBS; **(c)** Quantification of αSMA expression normalized to GAPDH; **(d)** Western blot analysis of p-p70 and p-S6 in colon lysates, **(e & f)** Quantification of p-p70 and p-S6 levels in colon lysates normalized to their total un-phosphorylated forms. Data are representative or cumulative ± SEM (t test) of n=3-5 mice/group from n=3 experiments $^{*}p < 0.05$, $^{**}p < 0.01$, $^{***}p < 0.001$.

Supplementary Figure 2

(a) Representative images of IHC showing a significant increase of trichrome blue staining and αSMA-positive staining in submucosal layers in active CD. **(b)** Quantification of trichrome blue staining and thickness of αSMA-positive layer. **(c)** Western blot analysis of αSMA expression and quantification (d). (e) qPCR analysis of fibrosis markers and cytokines. Data are presented as Mean ± SEM. n=3-4/group. $^{*}p < 0.05$, $^{**}p < 0.01$, $^{***}p < 0.001$ (ANOVA).

Supplementary Figure 3

(a) *Evaluation of cytokines IL-23, IL-22, and IL-1β by ELISA.* **(b)** Colonic lamina propria cells were prepared from mice treated with vehicle, rapamycin (Rapa), TNBS, and TNBS plus rapamycin. FACS analysis of $Cx3cr1^{+}$ resident mononuclear phagocytes. $Cx3cr1^{+}$ cells were further gated with macrophage activation markers, including MHC-II, CD40, CD80, and CD86; **(c)** Purification of CX3Cr1 cells in colon single-cell suspension using Miltenyi magnetic beads. **(d)** The purity of cells was further verified by FACS analysis using CD11b. About 92.2% of $Cx3cr1^{+}$ cells were CD11b-positive. Data are presented as mean ± SEM. n=4, $^{*}p < 0.05$, $^{**}p < 0.01$, $^{***}p < 0.001$.

Supplementary Figure 4

FACS analysis of CD64⁺/CD11c⁻/Cx3cr1⁺ cells. ***(a)*** *Gating strategy for analysis of CD64⁺/CD11c⁻/Cx3cr1⁺ cells.* ***(b)*** *P1-P4 sub-populations of Cx3cr1⁺ cells and quantification* ***(c)****.* ***(d)*** *qPCR quantification of cytokines in FACS-sorted populations.*

*Data are presented as mean ± SEM. n=3-4, *p< 0.05, **p< 0.01, ***p< 0.001.*

Supplementary Figure 5

(a) Schematic diagram showing the weekly TNBS treatment; **(b)** Representative images of mouse colon harvested on week 6 post-TNBS treatment; **(c)** Average length of the distal part of the colon, called 'left colon' (n=5-7 mice per group); **(d)** Cytokines detected by ELISA. **(e)** Lamina propria preparations from mouse colons were stimulated for 3 h with PMA/Ionomycin, followed by intracellular staining of cytokines for IL-23 and IL-1β. Cells were gated with $CD11b^+$ and $F4/80^+$ and then with IL-23 and IL-1β; **(f)** Quantification of the populations of $CD11b^+/IL\text{-}23^+$ and $CD11b^+/IL\text{-}1\beta^+$ in mouse colons. **(g)** qPCR analysis of cytokines. Data are represented as mean ± SEM. n=4-7 mice each group. *p< 0.05, **p< 0.01, ***p< 0.001.

Supplementary Figure 6

(a) Representative images of colon harvested from RAG KO mice (n=4-5 mice/group) on week 6 post-TNBS and/or rapamycin treatment and average length of the left colon samples **(b).**

Supplementary Figure 7

(a) Single-cell suspension prepared from colonic lamina propria stimulated with IL-23 (10 ng/ml) for 6 h in the presence of 1 μg/ml anti-IL22 neutralizing antibody. Western blot analysis of αSMA and **(b)** quantification of αSMA expression. **(c)** Representative images of αSMA staining and **(d)** quantification of the thickness of αSMA^+ layer. qPCR analysis of αSMA and Col I expression **(e)** and cytokines **(f)**. qPCR analysis of IL-22 and IL-17 expression after neutralization of IL-23 **(g)**. Data are presented as mean ±SEM. n=3-5. *p< 0.05, **p< 0.01, ***p< 0.001.

Supplementary Figure 8

Neutralization of IL-23 and IL-22 attenuates intestinal fibrosis in Cx3cr1Atgf/f mice. qPCR quantification of fibrosis markers (a) and western blot analysis of αSMA (b & c). Representative images of trichrome blue staining for collagen deposition (d) and quantification of collagen deposition (e). FACS analysis of αSMA-positive cells and quantification (f). Data are presented as mean ±SEM. n=3-4. *$p< 0.05$, **$p< 0.01$, ***$p< 0.001$.

Supplementary Figure 9

Depletion of CD90 cells attenuates intestinal fibrosis in RagKO mice. **(a)** Schematic diagram showing the weekly TNBS treatment and IP injection of CD90 neutralization antibody every other day; **(b)** FACS analysis of depletion of $CD90^{+}$ cells and quantification **(c)**. **(d)** Representative images of trichrome blue staining of collagen deposition and quantification (e). qPCR analysis of αSMA, Col-I, and Col-III **(f)**. Western blot analysis of αSMA **(g)** and quantification **(h)**. **(i)** FACS analysis of αSMA^{+} cells and quantification **(j)**. Data are presented as mean ± SEM. n=3-4 mice in each group. *$p< 0.05$, **$p< 0.01$, ***$p< 0.001$.

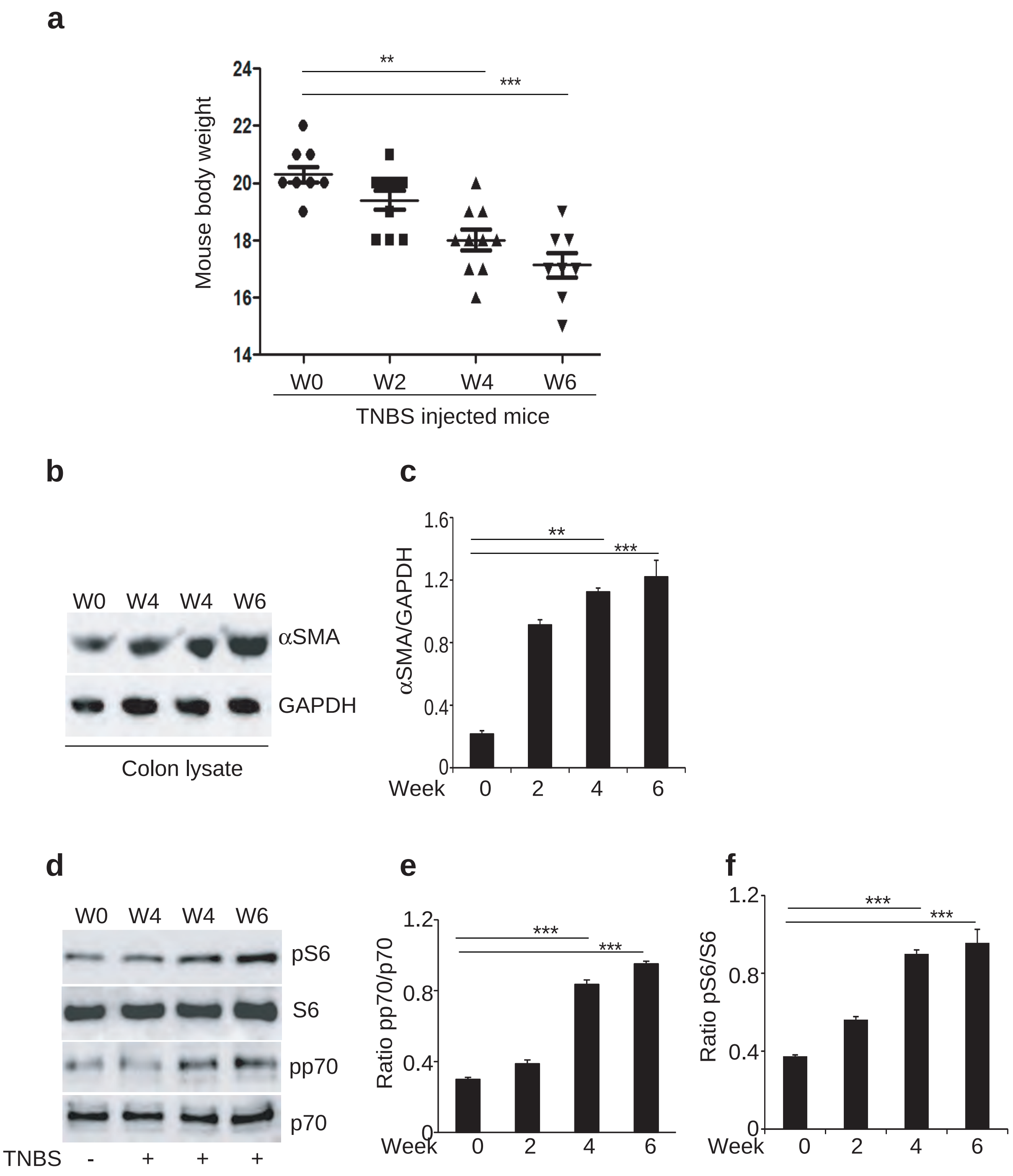

Revised Supplementary Figure 01

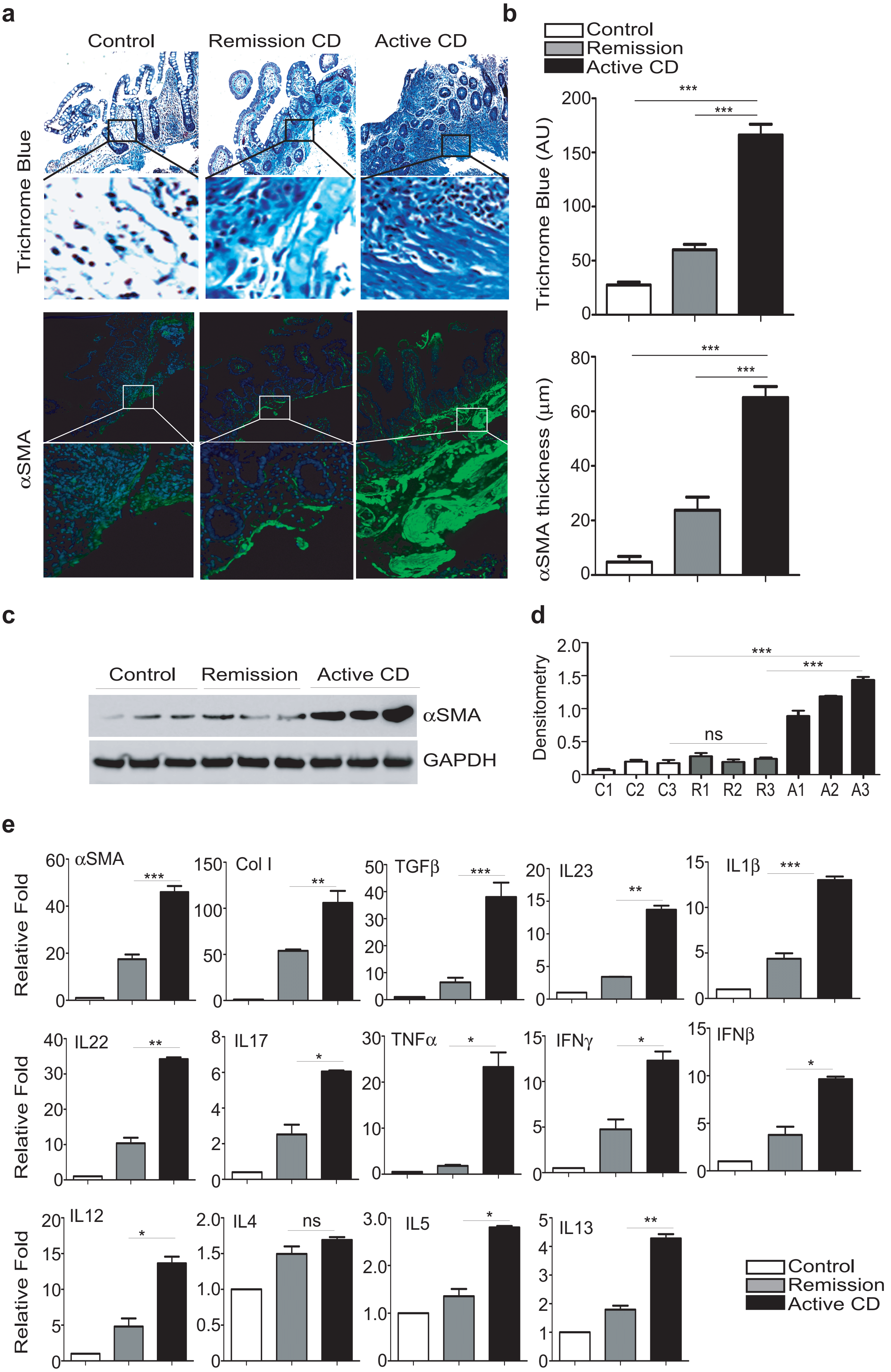

Revised Suplementary Figure 2

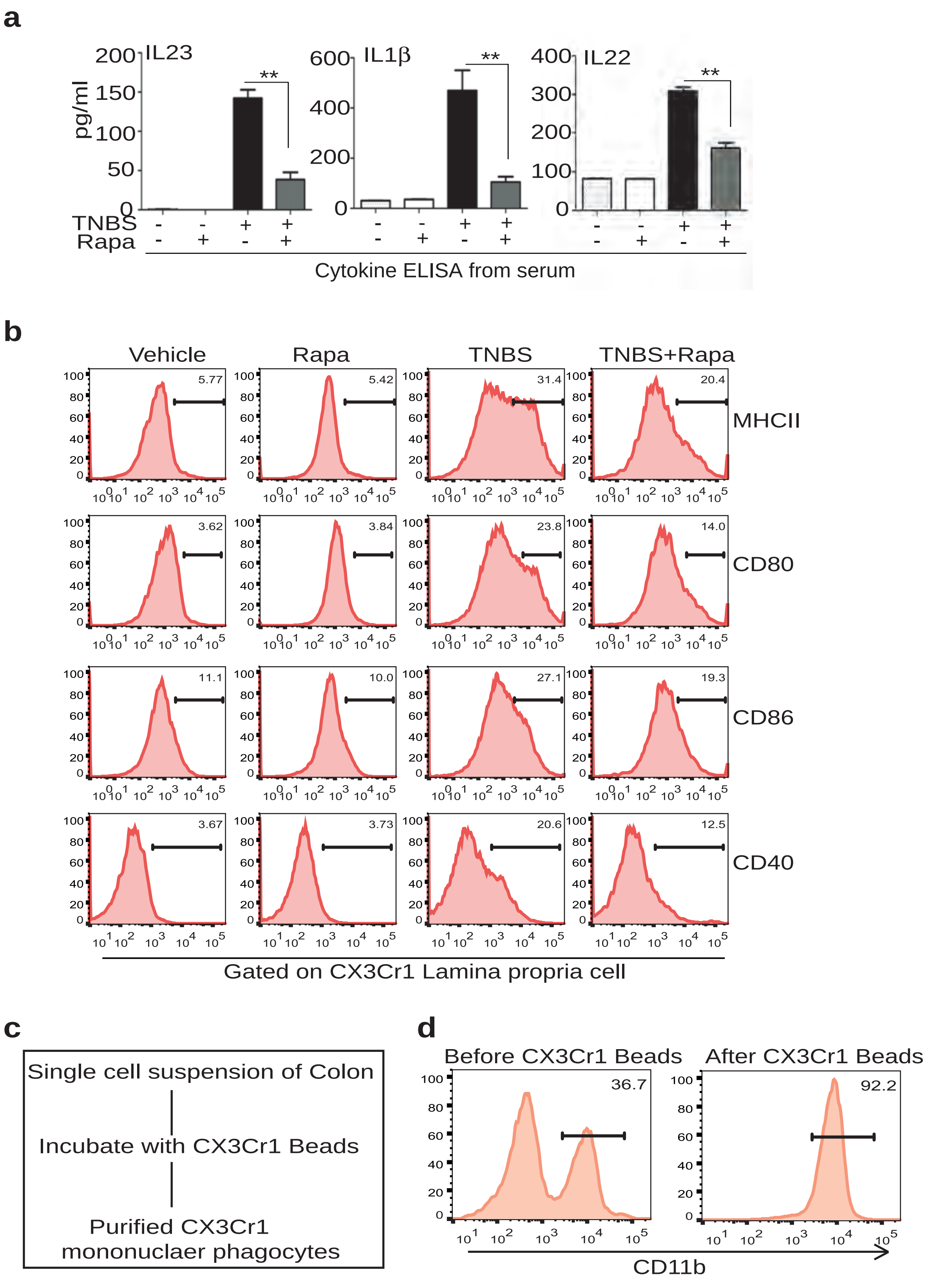

Revised Supplementary Figure 3

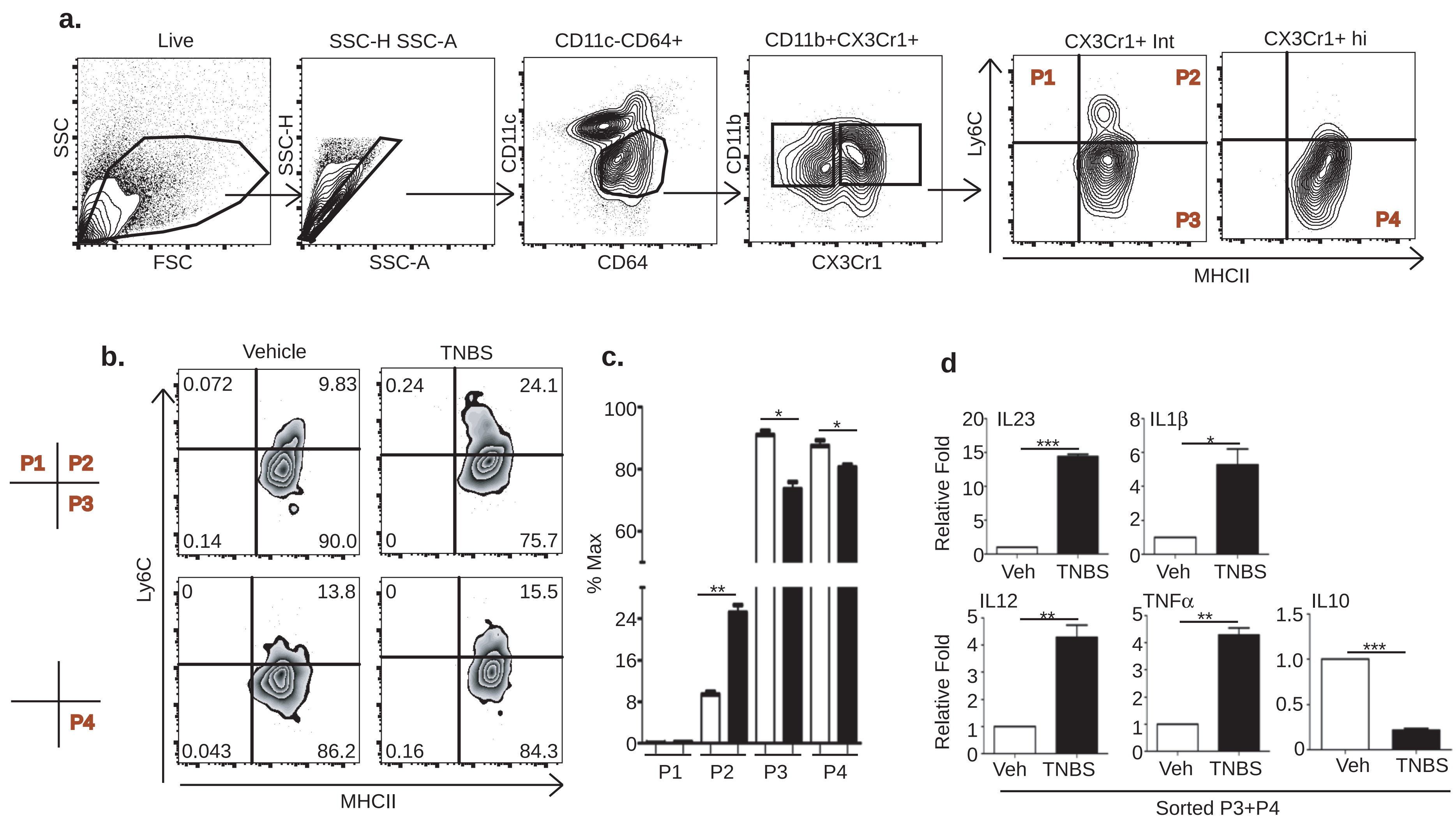

Revised Supplementray Figure_04

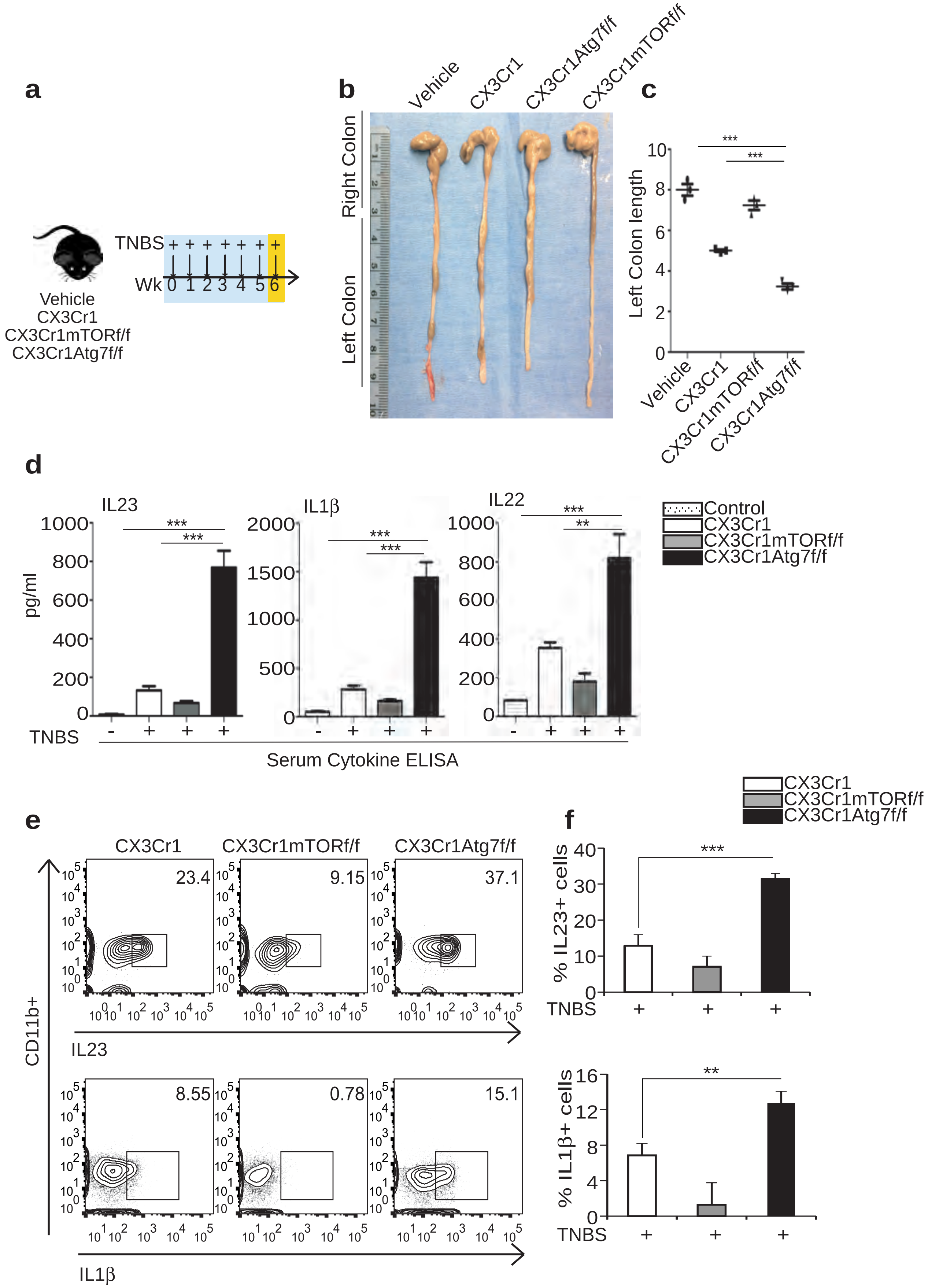

Revised Suplementary Figure 5a

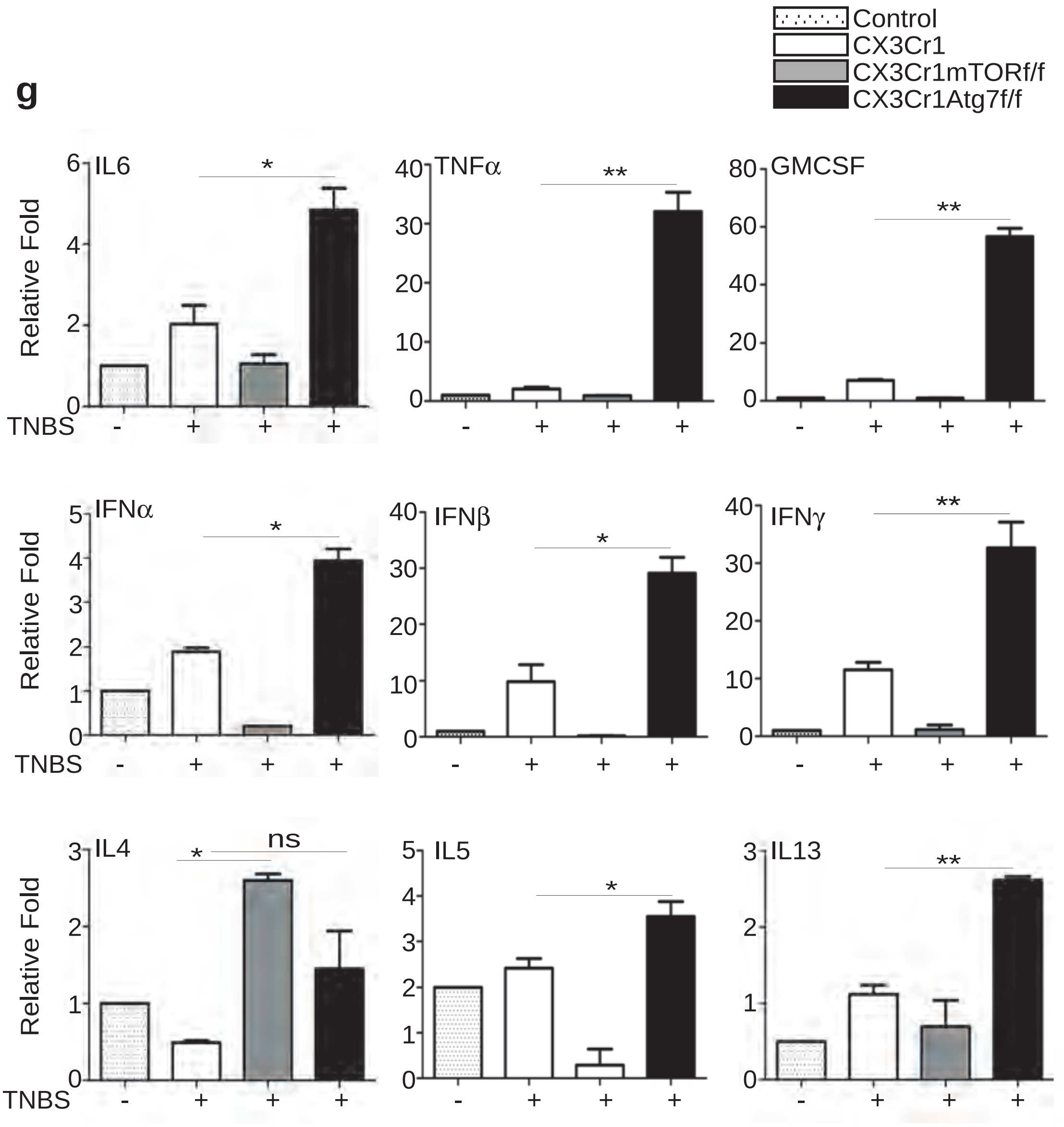

Revised Suplementary Figure 5b

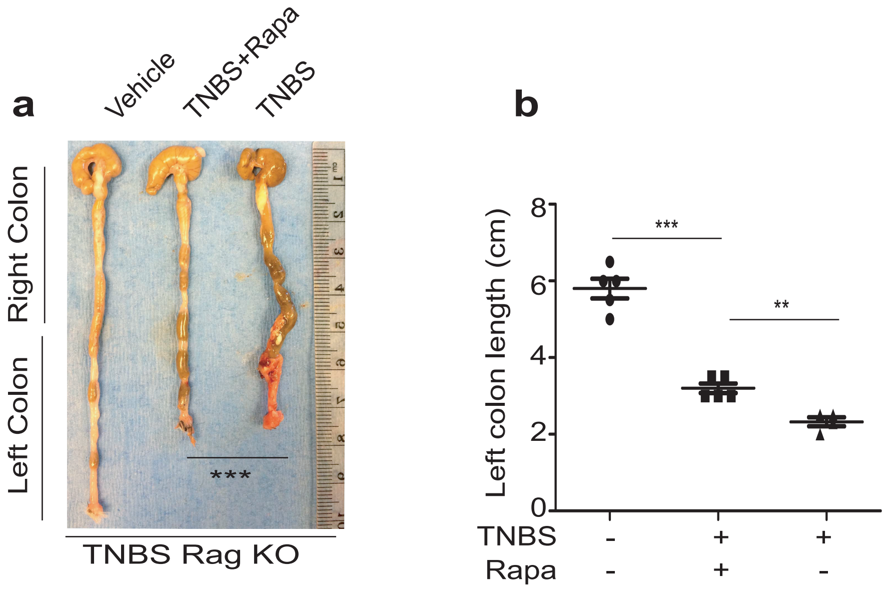

Revised Suplementary Figure 6

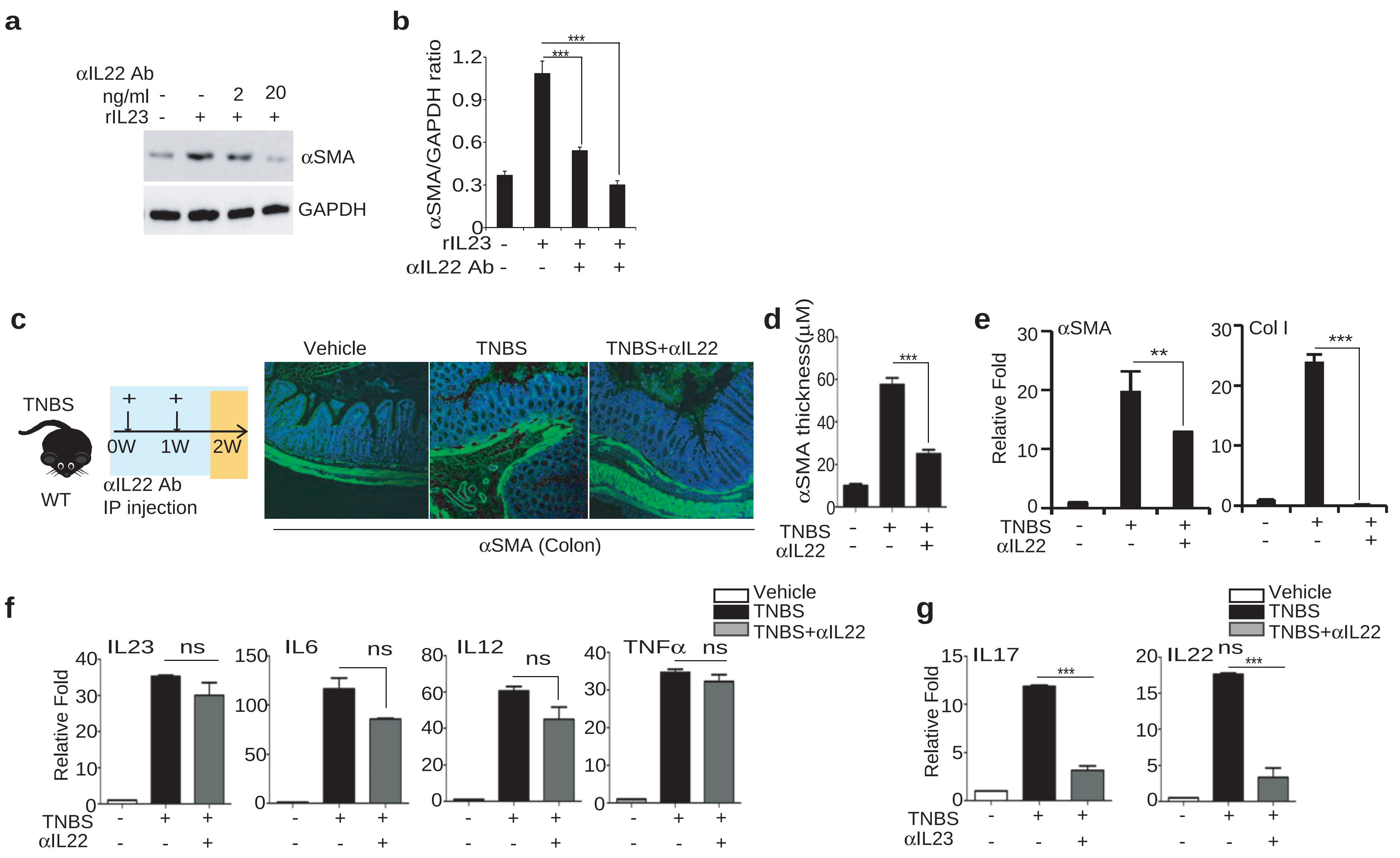

Revised Suplementary Figure 7

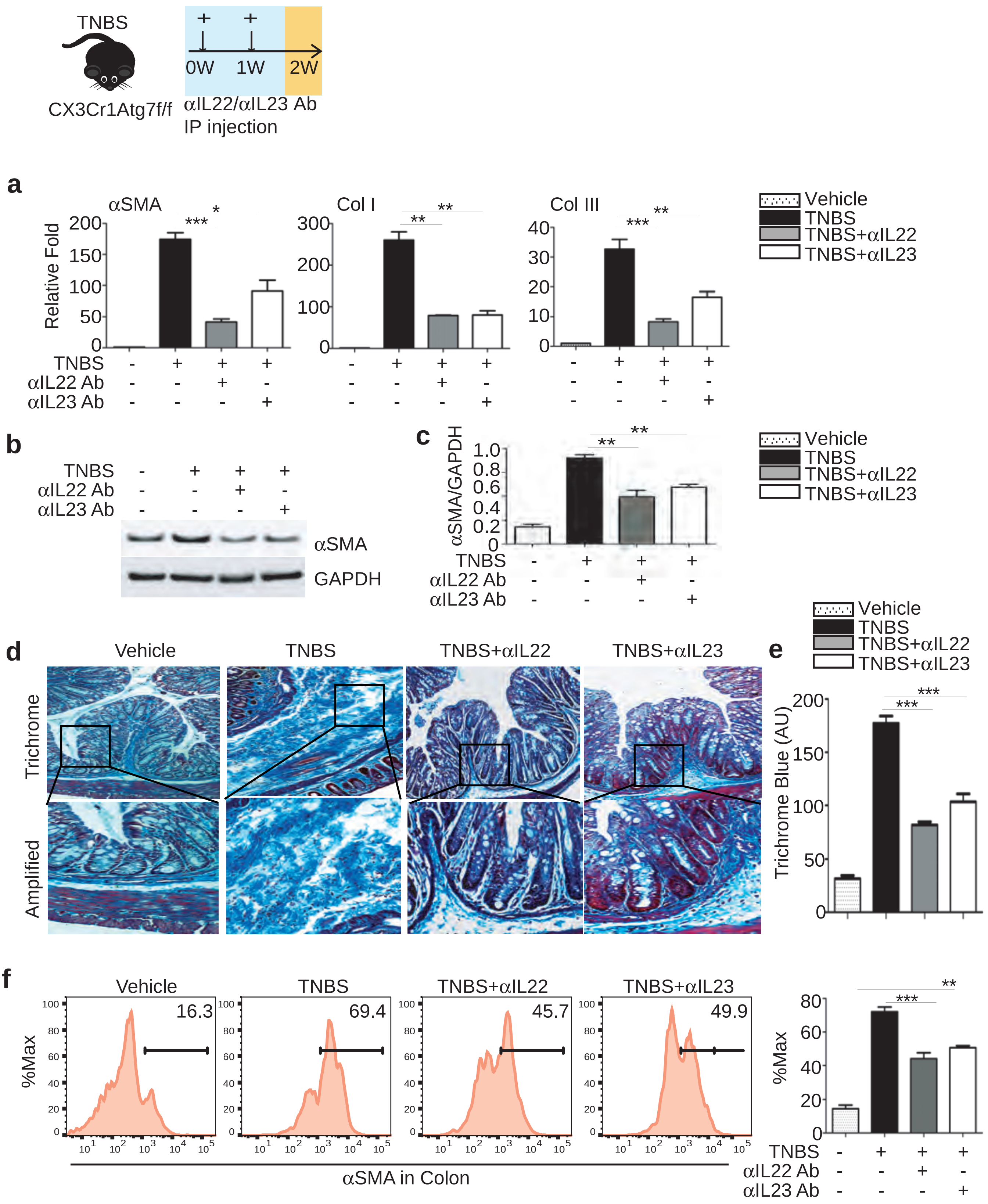

Revised Suplementary Figure 8

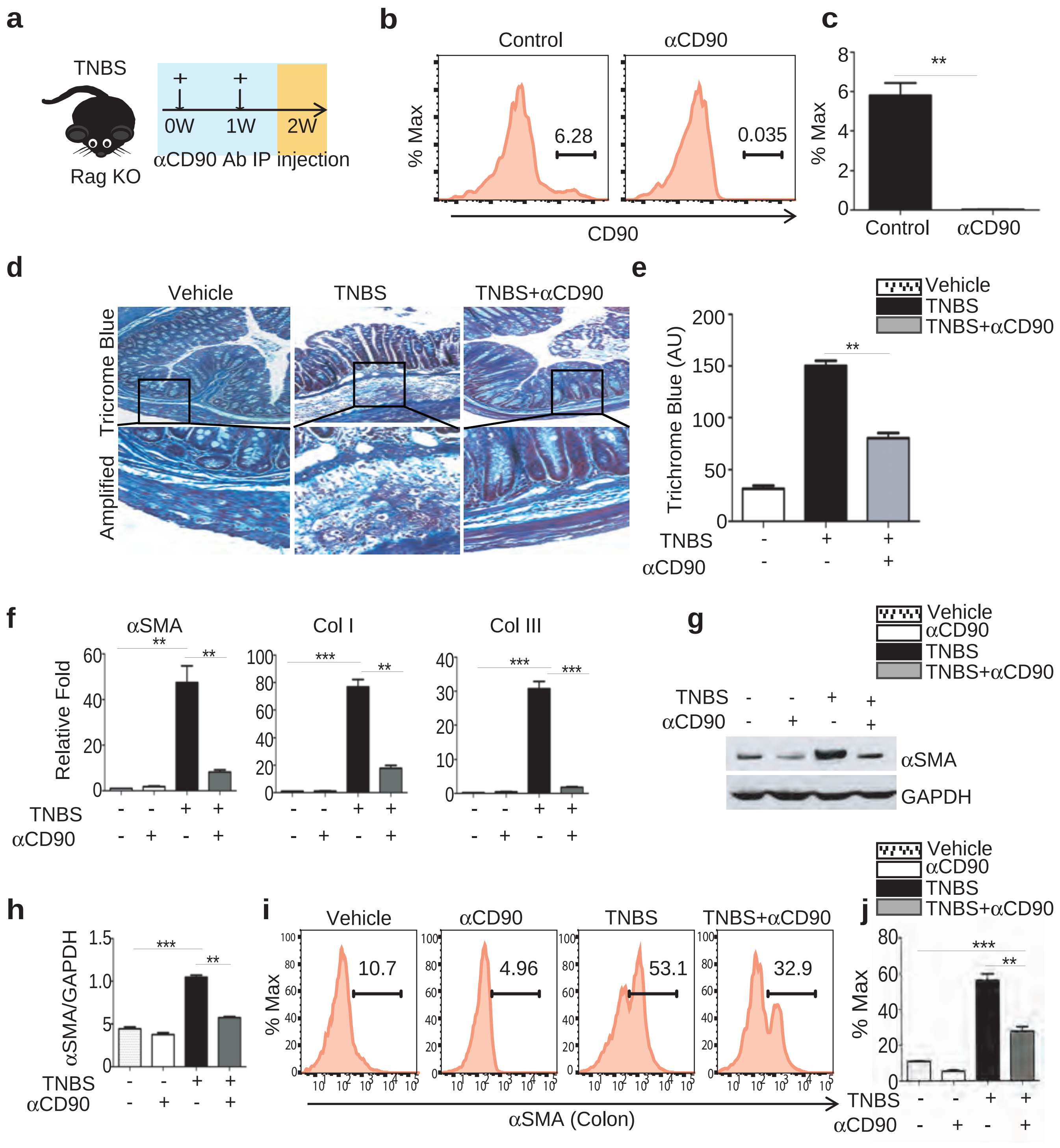

Revised Supplementary Figure-9
